# Dipole‐Manipulated Built‐In Electric Field Enables Ultrafast Charge Separation in Perylene Diimide Polymers for Photoelectrochemical Water Splitting

**DOI:** 10.1002/advs.75610

**Published:** 2026-05-07

**Authors:** Ying‐Xin Qiao, Zicong Situ, Yi‐Jing Chen, Shuo‐Xiang Liu, Jing‐Lan Zhang, Xingqing Li, Luo‐Han Xie, Si‐Hang Xie, Qing‐Xiao Tong, Andong Xia, Zhuoran Kuang, Jing‐Xin Jian

**Affiliations:** ^1^ Department of Chemistry Key Laboratory for Preparation and Application of Ordered Structural Materials of Guangdong Province Shantou University Guangdong P. R. China; ^2^ State Key Laboratory of Information Photonic and Optical Communications and School of Science Beijing University of Posts and Telecommunications Beijing P. R. China

**Keywords:** charge separation, light‐harvesting, perylene diimide, photoelectrochemistry, water splitting

## Abstract

Molecular dipole engineering in dendrimeric perylene diimide (PDI) polymers creates powerful built‐in electric fields (BIEFs) that dramatically enhance photoelectrochemical performance. By strategically tuning nitrogen content in aromatic linkers (pyridine, pyrimidine, and 1,3,5‐triazine), the pyridine‐linked C_5_N_1_‐PDI achieves exceptional photocurrent density (68.7 µA cm^−^^2^ at 1.23 V vs. RHE), outperforming its counterparts by 3.42–229 times. Ultrafast spectroscopy reveals this enhancement originates from sub‐picosecond charge separation and efficient multi‐electron accumulation, while theoretical modeling confirms the critical role of linker polarity in BIEF amplification. This work establishes a fundamental structure‐kinetics relationship for designing high‐performance organic photoelectrodes, providing a versatile strategy for advancing solar energy conversion technologies.

## Graphical abstract

Molecular dipole engineering in dendritic perylene diimide polymers through nitrogen‐rich heterocyclic linkers amplifies the built‐in electric field, enabling sub‐picosecond charge separation and efficient multi‐electron accumulation. This structure–dynamics–performance relationship establishes a versatile design strategy for high‐performance organic photoelectrodes in solar water splitting.

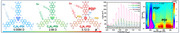

## 1 Introduction

Natural photosynthesis represents the most essential chemical process on Earth [1], sustaining life through solar‐driven carbon fixation to produce organic biomass and water oxidation to release O_2_ [2, 3]. This remarkable system relies on sophisticated light‐harvesting complexes (LHCs) [4, 5] that efficiently capture and directionally transfer solar energy to reaction centers, where charge separation initiates catalytic reactions [6]. Particularly crucial is the oxygen‐evolving complex (OEC) in Photosystem II, which catalyzes the challenging four‐electron water oxidation reaction through precisely orchestrated multi‐electron accumulation processes [7, 8]. Inspired by this natural blueprint, developing artificial photosynthetic systems for solar water splitting has emerged as a promising sustainable energy strategy [9]. The oxygen evolution reaction (OER) presents the fundamental bottleneck in artificial photosynthesis [10], requiring catalysts that can accumulate multiple holes and manage the complex proton‐coupled electron transfers while minimizing energy losses [11, 12, 13].

Among organic semiconductors, perylene diimides (PDIs) and their derivatives have garnered significant attention as photoanode materials due to their exceptional photostability and strong visible‐light absorption [14, 15, 16, 17]. However, their practical application in photoelectrocatalysis remains hampered by rapid charge recombination, short exciton diffusion lengths, and particularly the inability to efficiently sustain the multi‐hole accumulation essential for OER [18, 19, 20]. Generating a robust built‐in electric field (BIEF) presents a compelling strategy to address these limitations, as it can drive charge separation and direct transport through band bending and interfacial space charge effects [21]. The strength of such BIEF depends critically on both structural crystallinity and molecular dipole moment, the former enabling efficient charge transport through ordered pathways, while the latter facilitates exciton dissociation and generates the driving force for charge separation [22, 23, 24].

Previous approaches to enhance PDI performance have focused on supramolecular assembly or composite formation [25, 26, 27]. However, these systems typically suffer from limited long‐range order and insufficient dipole moments, resulting in inadequate charge separation driving force and poor stability under operational conditions. Covalent polymerization of PDI units offers a more robust alternative, providing enhanced intermolecular interactions and improved charge transport characteristics [28, 29, 30, 31, 32]. Seminal work by Zhu and coworkers demonstrated that crystalline PDI polymers could achieve notable OER activities [33], while our group previously established that strategic linker engineering could amplify molecular dipoles and BIEF strength, boosting photoelectrocatalytic performance by over two orders of magnitude compared to self‐assembled monomers [34]. Despite these advances, the fundamental mechanisms governing multi‐electron accumulation and the ultrafast charge transfer processes in such designed polymer systems remain poorly understood, particularly how molecular‐scale dipole alignment influences charge separation dynamics and OER efficiency [35, 36, 37, 38].

**SCHEME 1:**
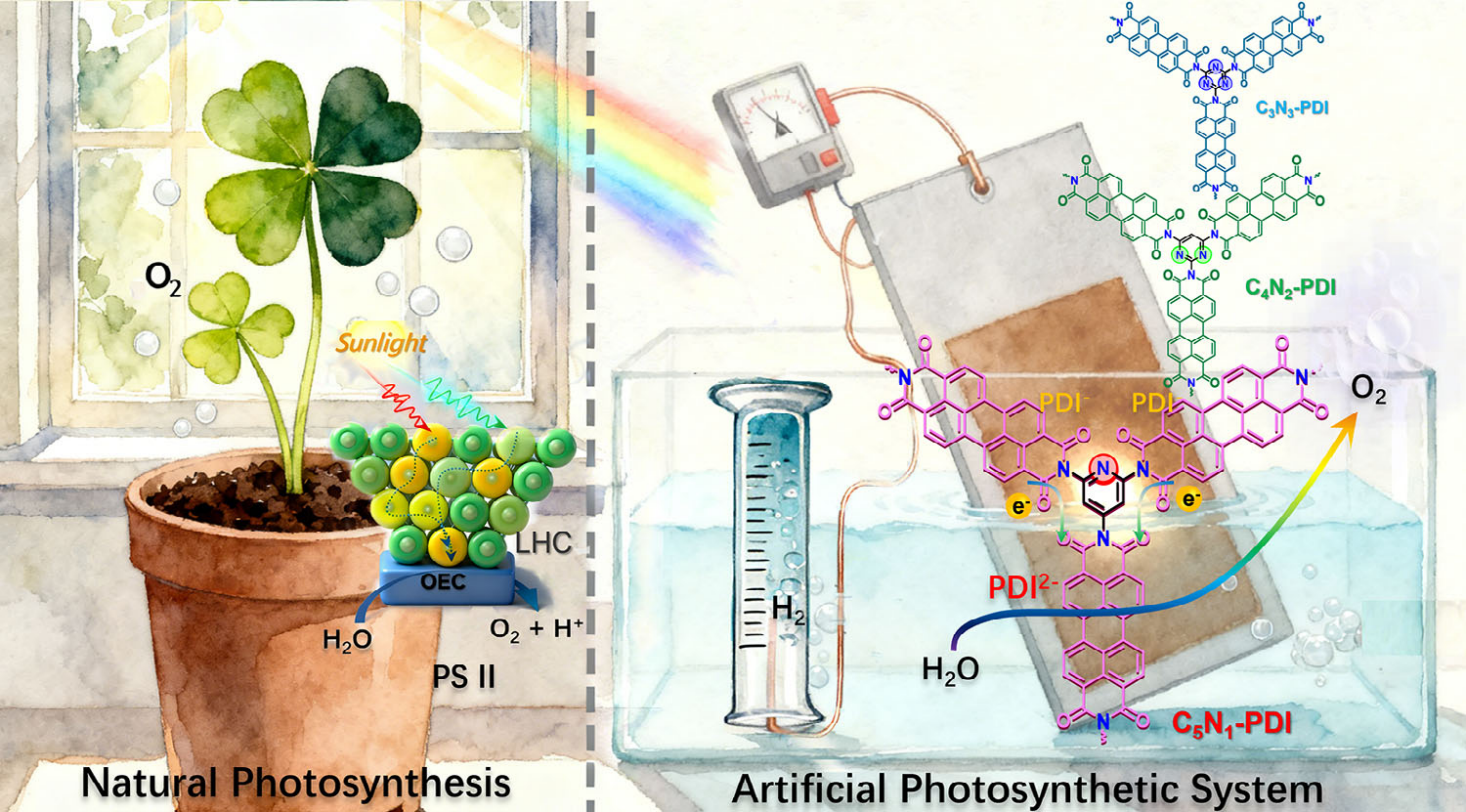
Biomimetic design and schematic illustration of the dendrimeric PDI polymers for PEC water splitting, mimicking the natural light‐harvesting complex (LHC) in Photosystem II.

Herein, we report a systematic investigation of dendrimeric PDI polymers featuring nitrogen‐regulated linkers—pyridine (C_5_N_1_‐PDI), pyrimidine (C_4_N_2_‐PDI), and 1,3,5‐triazine (C_3_N_3_‐PDI)—designed to mimic the hierarchical energy funneling of natural LHCs while enhancing multi‐electron accumulation capability for OER (Scheme 1). Through precise control of linker electronic structure, we verify that enhanced dipole strength significantly promotes ultrafast charge separation and multi‐electron accumulation—key processes overcoming the efficiency bottleneck in photoelectrochemical water oxidation. Combined ultrafast spectroscopy and theoretical calculations establish a direct correlation between linker polarity, BIEF magnitude, and charge separation kinetics, providing molecular‐level insights for designing high‐performance organic photoelectrodes.

## 2 Results and Discussion

### 2.1 Synthesis and Computational Modeling

**FIGURE 1:**
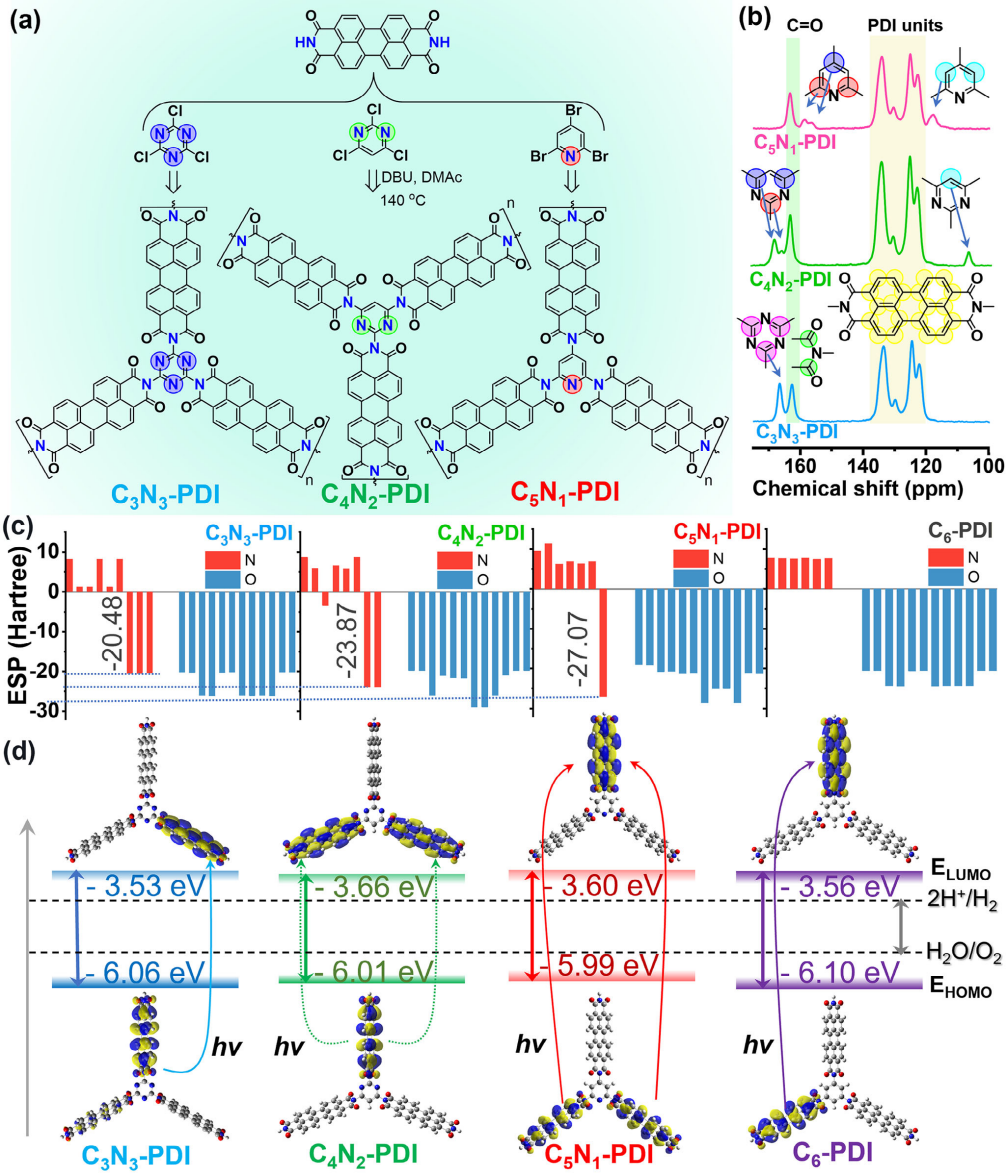
(a) Synthetic strategy (a) and solid‐state ^13^C NMR spectra (b) of C_5_N_1_‐PDI, C_4_N_2_‐PDI, and C_3_N_3_‐PDI polymers. (c) Calculated surface electrostatic potential (ESP) energies of key N and O atoms in the DFT‐optimized structures of C_5_N_1_‐PDI, C_4_N_2_‐PDI, C_3_N_3_‐PDI, and C_6_‐PDI. (d) Spatial distributions of HOMO and LUMO orbitals and corresponding energy level diagrams.

Figure 1a illustrates the synthetic routes for the C_5_N_1_‐PDI, C_4_N_2_‐PDI, and C_3_N_3_‐PDI polymers, utilizing 3,4,9,10‐perylenetetracarboxylic diimide (PDI) as the fundamental building block and 2,4,6‐tribromopyridine, 2,4,6‐trichloropyrimidine, and cyanuric chloride as the respective linking units [39]. Successful synthesis was confirmed by ^1^ ^3^C solid‐state NMR spectroscopy. As shown in Figure 1b, the carbon atoms of the PDI conjugate units in all three polymers exhibited signals in the chemical shift range of 122–135 ppm, while the carbonyl carbon atoms resonated in the lower‐field region of 162–163 ppm. Notably, the carbon atoms of the 1,3,5‐triazine linker resonated at 166.6 ppm [39], those of the pyrimidine linker at 168.3, 166.1, and 106.3 ppm, and those of the pyridine linker at 158.1, 155.8, and 117.2 ppm, further corroborating the successful formation of the target polymeric structures. Fourier‐transform infrared (FTIR) spectroscopy further validated the polymer structures, revealing C═O stretching vibration bands (ν_C═O_) at 1687, 1684, and 1687 cm^−^^1^ for C_5_N^1^^−^PDI, C_4_N_2_‐PDI, and C_3_N_3_‐PDI, respectively (Figure S1). Additionally, aromatic C_sp_^2^^−^H stretching vibrations (ν_Csp_^2^^−^_H_) from both the PDI units and the linking groups were observed, confirming the successful incorporation of the PDI moieties. Thermogravimetric analysis (TGA) demonstrated the excellent thermal stability of all three PDI polymers (Figure S2).

X‐ray photoelectron spectroscopy (XPS) was employed to determine the elemental composition of the polymeric materials, confirming the presence of C, N, and O elements and the absence of any residual metal species (Figure S3a). The nitrogen content in C_5_N_1_‐PDI, C_4_N_2_‐PDI, and C_3_N_3_‐PDI was determined to be 6.95%, 7.27%, and 7.42%, respectively. This increasing trend correlates directly with the rising number of nitrogen atoms in their respective linking groups and is consistent with elemental analysis results (Table S1). Deconvolution of the high‐resolution C 1s, O 1s, and N 1s XPS spectra provided further structural insights. The C 1s spectra were fitted with two‐component peaks, attributed to weakly polar C═C bonds and strongly polar C═O/C─N bonds (Figure S3b). The high‐resolution O 1s spectra exhibited two fitted peaks corresponding to O═C and O─H functional groups (Figure S3c). The primary feature in the N 1s spectra was assigned to the N═C species (Figure S3d), collectively providing compelling evidence for the successful formation of the PDI‐based polymers.

Density functional theory (DFT) calculations were performed to optimize the structural units of C_3_N_3_‐PDI, C_4_N_2_‐PDI, C_5_N_1_‐PDI, and a reference C_6_‐PDI (with a benzene linker) and to analyze their surface electrostatic potential (ESP) distributions. As illustrated in Figure S4, the negative charge (electron‐rich regions) in the PDI polymers is predominantly localized on the linking groups and the carbonyl oxygen atoms, while the positive charge is delocalized across the PDI conjugate backbone. Comparing the ESP energy barriers of the N and O atoms (Figure 1c), the pyridinic N in the C_5_N_1_‐PDI structure exhibits the lowest ESP value of −27.07 Hartree, significantly lower than that of the pyrimidine N in C_4_N_2_‐PDI (−23.87 Hartree) and the triazine N in C_3_N_3_‐PDI (−20.48 Hartree). Furthermore, the specific distribution of N within the pyrimidine and pyridine linkers results in substantially enlarged molecular dipole moments (µ) for C_4_N_2_‐PDI and C_5_N_1_‐PDI, reaching 2.66 and 3.12 D, respectively (Figure S4). These values are markedly greater than the nearly negligible dipole moments of the C_3_N_3_‐PDI (0.0084 D) and C_6_‐PDI (0.0077 D) polymers. The enhanced µ strengthens intermolecular interactions, which favor the formation of ordered stacking structures in the PDI polymeric materials, thereby promoting improved light‐harvesting efficiency and charge transport properties.

DFT calculations were also performed to determine the frontier molecular orbital distributions and energy level alignments of the different PDI polymer units. As shown in Figure 1d, the three PDI units are equivalent in both C_3_N_3_‐PDI and C_6_‐PDI, with the HOMO and LUMO spatially separated and distributed across different PDI conjugate rings. In C_4_N_2_‐PDI, the HOMO is localized on the PDI unit connected to the 2‐position of the pyrimidine ring, whereas the LUMO is delocalized over the two PDI units linked at the 4‐ and 6‐positions. In contrast, for C_5_N_1_‐PDI, the HOMO resides on the two PDI units at the ortho position to the pyridinic N, while the LUMO is localized on the single PDI unit at the position *para* to the pyridinic N. This spatial separation of the HOMO and LUMO implies the feasibility of intramolecular spatial charge transfer from one PDI unit to another upon photoexcitation. It is particularly noteworthy that C_5_N_1_‐PDI exhibits two distinct directional excitation transition pathways. This configuration is anticipated to funnel captured energy or charge toward the single PDI unit positioned *para* to the pyridinic N, thereby facilitating the formation of a multi‐electron accumulated state (e.g., PDI^2−^) and ultimately enabling highly efficient light harvesting and photoelectronic conversion.

### 2.2 Porous Structure and Photophysical Properties

The morphologies of the C_5_N_1_‐PDI, C_4_N_2_‐PDI, and C_3_N_3_‐PDI nanoparticles were investigated by field‐emission scanning electron microscopy (FE‐SEM) and high‐resolution transmission electron microscopy (HRTEM). Both C_4_N_2_‐PDI and C_3_N_3_‐PDI assembled into uniform nanosheets with widths below 200 nm, whereas C_5_N_1_‐PDI formed well‐defined nanospheres with diameters less than 50 nm (Figure S5). HRTEM images confirm the nanosheet‐like morphology with uniform particle sizes, and lattice fringe patterns are clearly observable at the 20 nm scale, providing direct evidence for local ordering and improved structural characterization (Figure S6). The uniform dimensions and well‐ordered morphologies observed for all three PDI polymers reflect their high crystallinity, which is advantageous for charge transport in photoelectrochemical applications.

**FIGURE 2:**
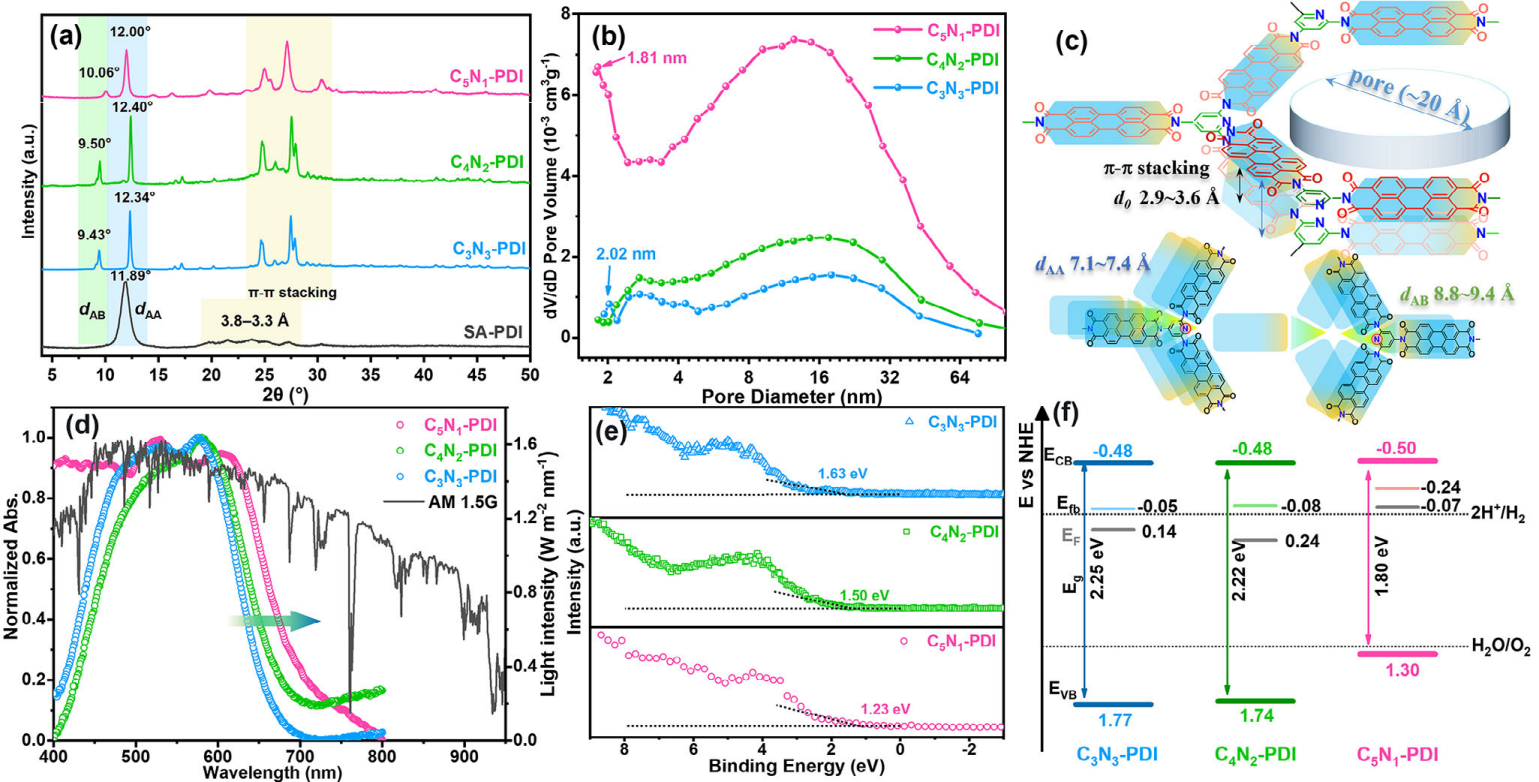
(a) High‐resolution XRD patterns of C_5_N_1_‐PDI, C_4_N_2_‐PDI, C_3_N_3_‐PDI, and SA‐PDA, indicating crystalline order and *π–π* stacking distances. (b) Pore size distributions derived from BET measurements. (c) Schematic illustration of molecular stacking modes, defining face‐to‐face (d_AA_) and cross‐stacking (d_AB_) interlayer distances and intrinsic cavity sizes. (d) UV–vis absorption spectra of C_5_N_1_‐PDI, C_4_N_2_‐PDI, and C_3_N_3_‐PDI, overlaid with the AM 1.5G solar spectrum, demonstrating wide visible‐light harvesting. (e) XPS valence band spectra and (f) constructed energy level diagrams of C_5_N_1_‐PDI, C_4_N_2_‐PDI, and C_3_N_3_‐PDI, showing suitable band alignment for water oxidation.

The crystalline nature and molecular stacking modes of the PDI polymers were further elucidated by high‐resolution X‐ray diffraction (HRXRD) (Figure 2a), while their porous characteristics were evaluated using nitrogen sorption measurements (Figure 2b). As depicted in Figure 2a, self‐assembled PDI (SA‐PDI) exhibited multiple diffraction peaks in the 20°–30° range, corresponding to *π–π* stacking distances between 3.30 and 3.80 Å, along with a peak at 11.89° assigned to a face‐to‐face interlayer spacing (d_AA_) of 7.42 Å [40]. In contrast, the C_5_N_1_‐PDI, C_4_N_2_‐PDI, and C_3_N_3_‐PDI polymers showed sharper and shifted *π–π* diffraction peaks toward higher angles, indicating shortened *π–π* stacking distances and enhanced structural ordering. Specifically, the *π–π* stacking distances in C_3_N_3_‐PDI were determined as 3.60, 3.24, and 3.20 Å. These values decreased to 3.59, 3.23, and 3.18 Å in C_4_N_2_‐PDI, and further contracted to 3.56, 3.28, and 2.94 Å in C_5_N_1_‐PDI, underscoring progressively stronger interlayer *π–π* interactions, particularly in C_5_N_1_‐PDI, that facilitate efficient charge carrier transport.

In the low‐angle region (<20°), distinct diffraction peaks corresponding to face‐to‐face (d_AA_) and cross‐stacking (or slipped‐parallel, d_AB_) interlayer distances were observed [41]. Relative to SA‐PDI (d_AA_ = 7.42 Å), the d_AA_ spacing decreased from 7.42 Å in the less polar C_3_N_3_‐PDI to 7.13 and 7.16 Å in the strong polarity C_4_N_2_‐ PDI and C_5_N_1_‐PDI, respectively, indicating intensified interlayer interactions in polymers incorporating polar pyridine‐based linkers. Conversely, the d_AB_ spacing increased from 8.78 Å in C_3_N_3_‐PDI to 9.30 and 9.36 Å in C_4_N_2_‐PDI and C_5_N_1_‐PDI (Figure 2c), suggesting that enhanced molecular dipole polarity effectively modulates the cross‐stacking architecture, potentially optimizing carrier mobility and light‐harvesting performance.

The porous characteristics of the C_5_N_1_‐PDI, C_4_N_2_‐PDI, and C_3_N_3_‐PDI polymers were systematically investigated through N_2_ physisorption measurements. As illustrated in Figure S7, all three polymers exhibit Type IV adsorption isotherms with distinct H_2_‐type hysteresis loops, indicative of mesoporous structures. The Barrett‐Joyner‐Halenda (BJH) method revealed pore size distributions centered in the mesoporous region (Figure 2b) [42, 43]. Notably, the BET specific surface areas follow the order: C_5_N_1_‐PDI (77.37 m^2^ g^−1^) > C_4_N_2_‐PDI (24.50 m^2^ g^−1^) > C_3_N_3_‐PDI (14.10 m^2^ g^−1^) (Table S2). The substantially larger surface area of C_5_N_1_‐PDI aligns with its more compact packing architecture (Figure 2c), driven by enhanced µ and strengthened *π–π* interactions as established in our DFT calculations and HRXRD results. Furthermore, the minimum pore sizes range from 1.81 to 2.02 nm, consistent with the intrinsic cavity dimensions of the molecular frameworks. The combination of mesopores (5–30 nm) and high specific surface area collectively provides abundant accessible active sites, facilitating efficient mass transport and catalytic processes.

The light‐harvesting capabilities of the PDI polymers were evaluated by UV–vis absorption spectroscopy (Figure 2d). All materials display broad absorption spanning the 400–800 nm visible region, demonstrating significant spectral overlap with the AM 1.5 G solar spectrum. Particularly noteworthy is C_5_N_1_‐PDI, which exhibits a substantially redshifted absorption edge and enhanced absorption intensity across the visible range compared to its counterparts. Tauc plot analysis yields optical band gaps (E_g,opt_) of 1.84, 1.91, and 1.95 eV for C_5_N_1_‐PDI, C_4_N_2_‐PDI, and C_3_N_3_‐PDI, respectively (Figure S8), a trend that correlates well with the computed HOMO‐LUMO gaps from our DFT studies. Photoluminescence experiments reveal that all three polymer materials exhibit extremely low fluorescence quantum efficiencies (Table S3)—far below that of the PDI monomer in DMF solvent—indicating that, upon light absorption, the PDI polymers readily generate charge‐separated states prone to driving catalytic reactions.

To elucidate the electronic structure relevant to photoelectrocatalytic performance, we constructed energy level diagrams by integrating XPS valence band spectra (Figure 2e) and electrochemical data from cyclic voltammetry (CV) (Figure S9). The valence band‐to‐Fermi level differences (E_VB_‐E_F_) determined by XPS, combined with conduction band potentials (E_CB_) estimated from reduction peaks, reveal favorable band alignment for water oxidation. As depicted in Figure 2f, C_5_N_1_‐PDI possesses the narrowest band gap among the series, with a valence band position thermodynamically suited for driving the oxygen evolution reaction. Complementary Mott–Schottky measurements (Figure S10) confirm the formation of beneficial band bending at the electrode/electrolyte interface, which promotes the efficient separation and migration of photogenerated holes toward the electrolyte, thereby facilitating the OER process.

### 2.3 Photoelectrocatalytic Performance

**FIGURE 3:**
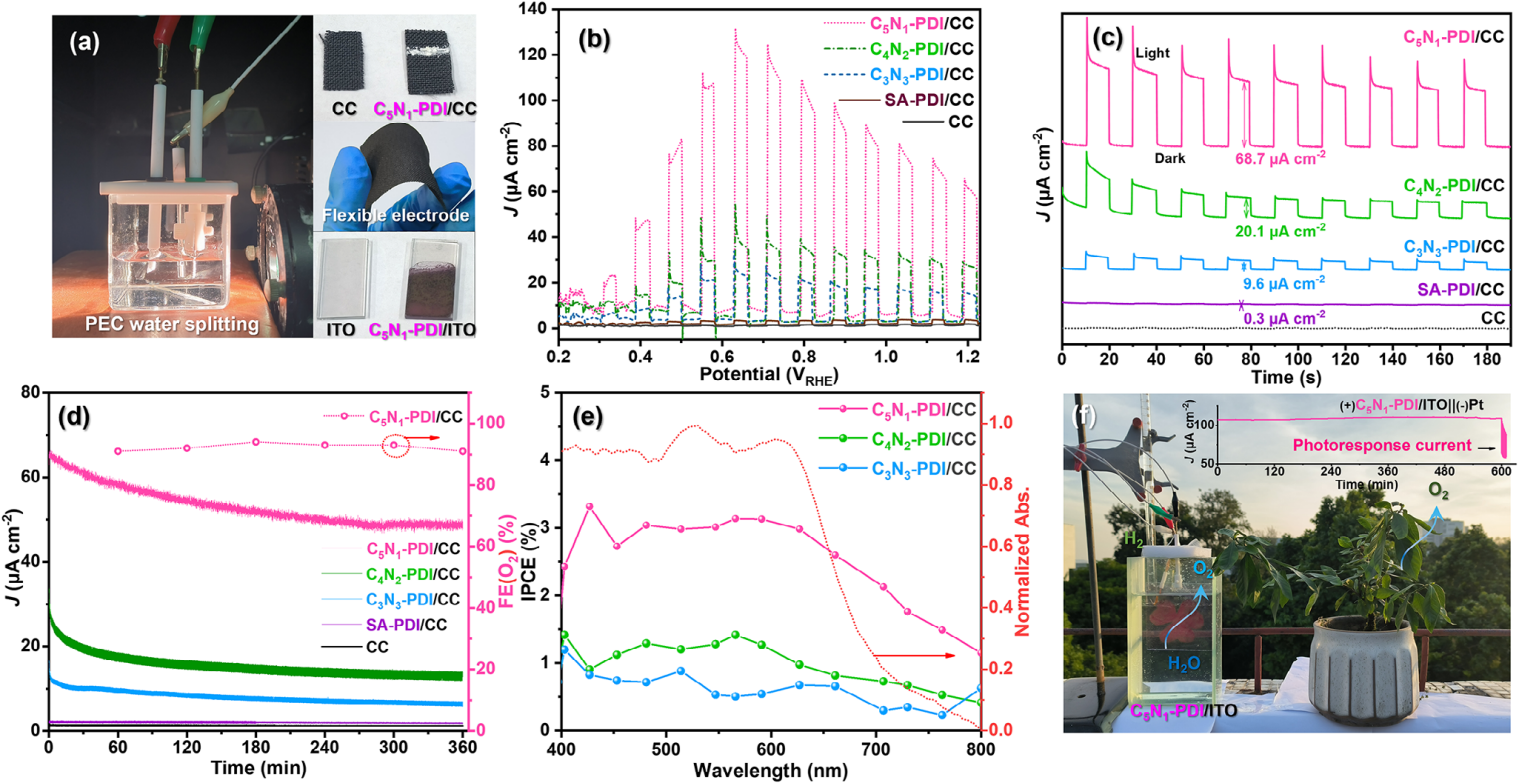
(a) Photographs of flexible carbon cloth and planar ITO electrodes coated with C_5_N_1_‐PDI polymer, and the schematic setup for PEC testing under simulated sunlight. (b) LSV curves under chopped AM 1.5G illumination, in 0.5 m Na_2_SO_4_ solution (pH 6.8), at a scan rate of 10 mV s^−1^. (c) Chronoamperometric (*i–t*) responses at 1.23 V vs. RHE under chopped light. (d) Long‐term stability over 360 min of continuous operation; Faradaic efficiency for O_2_ evolution was quantified by gas chromatography. (e) IPCE spectra correlated with the absorption profile of C_5_N_1_‐PDI. (f) Field test of the C_5_N_1_‐PDI/ITO photoanode under natural sunlight for artificial photosynthesis, with inset showing the photocurrent in a two‐electrode configuration at 1.2 V bias.

The PDI polymers could be readily deposited onto flexible carbon cloth (CC) or planar indium tin oxide (ITO) substrates, enabling their use in diverse device configurations (Figure 3a, Figure S11). Their photoelectrocatalytic water‐splitting performance was evaluated under simulated solar illumination (AM 1.5 G, 100 mW·cm^−2^) using CC‐supported electrodes. Linear sweep voltammetry (LSV) revealed that the C_5_N_1_‐PDI/CC electrode generated a substantially higher photocurrent density (j_ph_) within the potential range of 0.3–1.23 V vs. RHE, significantly outperforming the C_4_N_2_‐PDI, C_3_N_3_‐PDI, and self‐assembled PDI (SA‐PDI) references (Figure 3b). Notably, the C_5_N_1_‐PDI/CC electrode exhibited a low onset potential (E_onset_) at approximately 0.3 V vs. RHE, indicating favorable band alignment for efficient charge injection into the electrolyte. The photocurrent rose sharply with increasing bias, reaching a maximum exceeding 120 µA·cm^−2^ at 0.65 V vs. RHE—significantly higher than all other electrodes. The applied bias photon‐to‐current efficiency (ABPE) was calculated according to the equation ABPE = [*J*_ph_ × (1.23–V_app_) / P] × 100%, where *J*_ph_ is the photocurrent density at the applied bias (V_app_), and P is the incident illumination power density. The C_5_N_1_‐PDI/CC electrode achieved a maximum ABPE of ∼0.075%, substantially surpassing those of C_4_N_2_‐PDI/CC (0.025%), C_3_N_3_‐PDI/CC (0.016%), and SA‐PDI/CC (0.002%) (Figure S12).

Chronoamperometry (*i–t*) measurements under chopped illumination confirmed reproducible photocurrent generation, with transient features indicative of ongoing charge separation and recombination processes (Figure 3c). The optimal C_5_N_1_‐PDI electrode achieved a photocurrent density of 68.7 µA·cm^−^^2^ at 1.23 V vs. RHE, which is 3.42, 7.16, and 229 times greater than those of C_4_N_2_‐PDI/CC (20.1 µA·cm^−^^2^), C_3_N_3_‐PDI/CC (9.6 µA·cm^−^^2^), and SA‐PDI/CC (0.3 µA·cm^−^^2^), respectively. This performance places C_5_N_1_‐PDI among the top‐performing organic semiconductor photoanodes reported to date (Table S4) [12, 32, 33, 34, 44, 45, 46, 47, 48, 49, 50, 51, 52].

Furthermore, all PDI‐based electrodes exhibited excellent reproducibility and stability over 360 min of continuous illumination (Figure 3d). Under continuous illumination, the working and counter electrodes of the C_5_N_1_‐PDI/CC system generated visible oxygen and hydrogen bubbles. Gas chromatography confirmed oxygen evolution with a Faradaic efficiency exceeding 90% for the C_5_N_1_‐PDI/CC electrode, verifying that the photocurrent primarily originates from the oxygen evolution reaction. Incident photon‐to‐current efficiency (IPCE) spectra collected using monochromatic LED light showed that C_5_N_1_‐PDI delivers markedly higher efficiencies across the visible region compared to the other polymers (Figure 3e). The decline in IPCE beyond 700 nm for all samples aligns well with their respective optical absorption edges, underscoring the consistency between photo‐response and light‐harvesting capability.

To preliminarily assess practical applicability, a large‐area ITO electrode coated with C_5_N_1_‐PDI (>10 cm^2^) was tested under natural sunlight in a two‐electrode configuration of (+)C_5_N_1_‐PDI/ITO||Pt(−) in 0.5 m Na_2_SO_4_ solution (pH 6.8). At an applied bias of 1.2 V, the electrode continuously evolved both H_2_ and O_2_, maintaining a stable photoresponse current for over 600 min (Figure 3f). Post‐stability PXRD and FTIR analyses confirmed that the structural integrity and chemical composition of the polymer were well preserved after prolonged operation (Figure S13). This proof‐of‐concept demonstration highlights the potential of all‐organic PDI‐based materials in real‐world solar‐driven artificial photosynthesis and photoelectrocatalytic water‐splitting systems.

### 2.4 Interfacial Built‐in Electric Field and Charge Transfer Analysis

**FIGURE 4:**
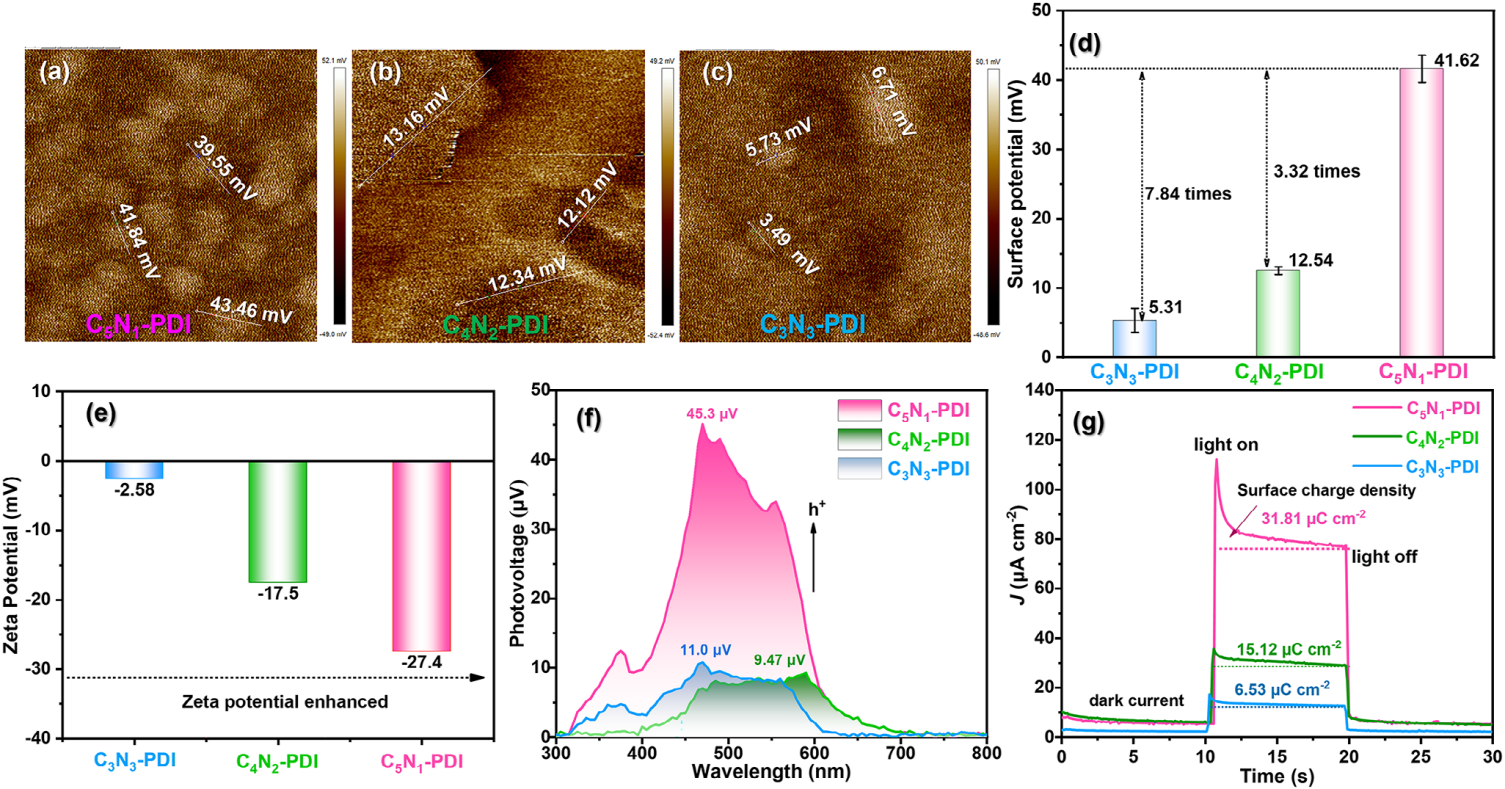
KPFM images and quantified surface potentials of C_5_N_1_‐PDI (a), C_4_N_2_‐PDI (b), and C_3_N_3_‐PDI (c). Comparison of their surface potential (d), Zeta potential (e), steady‐state surface photovoltage (f), and calculated surface charge density under illumination (g). Collectively, these data corroborate the strongest built‐in electric field in C_5_N_1_‐PDI.

The interfacial characteristics of the PDI polymers were systematically probed to elucidate the origin of their superior photoelectrochemical performance. Kelvin probe force microscopy (KPFM) measurements revealed significant differences in surface potential among the three materials (Figure 4a–c). C_5_N_1_‐PDI, organized as spherical nanoparticles, exhibited the highest average surface potential of 41.62 ± 1.96 mV, which is 3.32 times and 7.84 times greater than those of sheet‐like C_4_N_2_‐PDI (12.54 ± 0.54 mV) and C_3_N_3_‐PDI (5.31 ± 1.73 mV), respectively (Figure 4d). Complementary zeta potential analysis in aqueous solution showed negative surface charges for all three PDI nanoparticles, indicating a pronounced tendency to adsorb hydroxyl anions (OH^−^) that facilitates the oxygen evolution reaction, a characteristic feature of PDI‐based semiconductors. Notably, C_5_N_1_‐PDI displayed the most negative zeta potential of −27.4 mV, representing 1.57 and 10.62‐fold enhancements over C_4_N_2_‐PDI (−17.5 mV) and C_3_N_3_‐PDI (−2.58 mV), respectively (Figure 4e). These markedly enhanced surface properties directly reflect the strengthened built‐in electric field (BIEF) in C_5_N_1_‐PDI, consistent with its superior molecular polarity as established by our DFT calculations.

The interfacial BIEF serves as a crucial kinetic factor governing the direction and efficiency of charge separation and transfer. Its magnitude (F_BIEF_) can be quantitatively described by the relationship: F_s_ = (−2V_s_ρ/εε_0_)^1/2^, where V_s_ represents the surface potential, ρ denotes the surface charge density, ε is the low‐frequency dielectric constant, and ε_0_ is the vacuum permittivity. Since ε and ε_0_ remain essentially constant, the BIEF strength is primarily determined by V_s_ and ρ, which were experimentally assessed through surface photovoltage (SPV) spectroscopy and transient photocurrent measurements, respectively [33]. Steady‐state SPV spectra (Figure 4f) exhibited positive signals in the 400–600 nm range for all three polymers, with spectral profiles aligning well with their respective UV–vis absorption and IPCE characteristics. These positive SPV signals unequivocally indicate the accumulation of photogenerated holes at the material surface, suggesting efficient hole transfer under illumination that directly promotes oxidation reactions such as water splitting. Importantly, C_5_N_1_‐PDI yielded the largest SPV value of 45.3 µV, significantly surpassing C_4_N_2_‐PDI (9.47 µV), C_3_N_3_‐PDI (11.0 µV), and even the previously benchmarked Urea‐PDI (27.0 µV) [33].

The surface charge density (ρ), proportional to the number of accumulated positive charges, was determined by integrating the transient photocurrent density after subtracting steady‐state values over time. As depicted in Figure 4g, C_5_N_1_‐PDI achieved a maximum surface charge density of 31.81 µC·cm^−2^, which is 2.10 and 4.87 times greater than those of C_4_N_2_‐PDI (15.12 µC·cm^−2^) and C_3_N_3_‐PDI (6.53 µC·cm^−2^), respectively, under identical conditions. This result indicates that C_5_N_1_‐PDI possesses the most extensive space‐charge region, reflecting favorable band alignment and bending at the semiconductor‐electrolyte interface. Consequently, C_5_N_1_‐PDI exhibits the strongest BIEF, calculated to be 3.17 and 4.48 times greater than those of C_4_N_2_‐PDI and C_3_N_3_‐PDI, respectively. This enhanced electric field effectively promotes directional charge separation and transport across the interface. Further validation through electrochemical impedance spectroscopy (EIS) revealed a substantially smaller arc radius for the C_5_N_1_‐PDI electrode in the frequency range of 1–10^5^ Hz compared to its counterparts (Figure S14), indicating significantly reduced interfacial charge transfer resistance. This minimized resistance facilitates efficient charge carrier transport across the electrode‐electrolyte interface, providing a fundamental explanation for its high‐performance photoelectrochemical water splitting.

### 2.5 Ultrafast Charge Separation and Multi‐Electron Accumulation Dynamics

**FIGURE 5:**
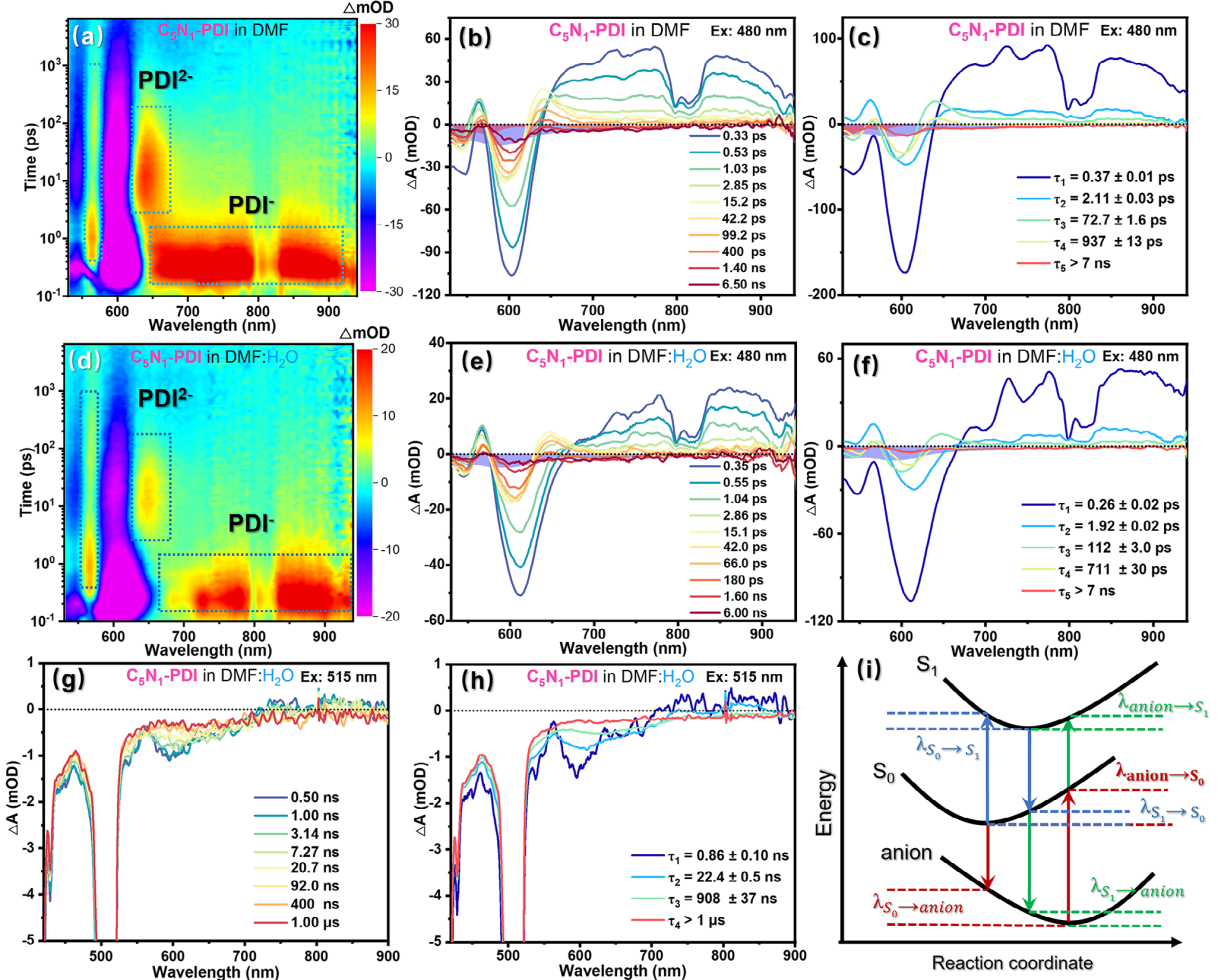
Ultrafast transient absorption (TA) spectroscopy reveals charge separation and multi‐electron accumulation dynamics. (a,d) 2D pseudocolor plots of fs‐TA spectra for C_5_N_1_‐PDI in (a) DMF and (d) DMF:H_2_O. (b,e) Corresponding temporal spectral evolution. (c,f) Evolution‐associated difference spectra (EADS) from global analysis. (g) Time evolution of ns‐TA spectra and (h) corresponding EADS in DMF:H_2_O, highlighting the decay of the two‐electron accumulated state (PDI^2^^−^). (i) Schematic diagram of transitions among ground state (S_0_), excited state (S_1_), and charged states, illustrating the kinetic pathway for efficient photoelectric conversion.

Efficient photoelectrochemical reactions rely critically on ultrafast charge separation (CS) and multi‐electron accumulation processes. To elucidate these fundamental pathways, we investigated the excited‐state dynamics of the PDI polymers using femtosecond (fs‐) and nanosecond transient absorption (ns‐TA) spectroscopy. Upon 480 nm excitation of C_5_N_1_‐PDI in DMF solution, the initial fs‐TA spectrum at 0.33 ps exhibits a ground‐state bleach (GSB) band centered around 600 nm and a broad excited‐state absorption (ESA) band spanning 650–950 nm (Figure 5a,b). This ESA feature closely resembles that of the PDI anion (PDI^−^) [53, 54], indicating ultrafast formation of a charge‐transfer (CT) state between adjacent PDI units immediately following photoexcitation. As the time delay increases to over 10 ps, a new species emerges with an ESA band around 620–650 nm, characteristic of the PDI dianion (PDI^2^^−^) [55, 56], accompanied by concurrent decay of the initial spectral features. The subsequent signal decrease to negative values reflects exciton dissociation and free carrier (FC) generation. This spectral evolution unequivocally demonstrates the occurrence of an ultrafast intramolecular CS process followed by multi‐electron accumulation and FC generation in C_5_N_1_‐PDI. Similar phenomena, though less pronounced, are observed in C_4_N_2_‐PDI and C_3_N_3_‐PDI (Figures S15 and S16). Notably, all these spectral characteristics are entirely distinct from those of SA‐PDI (Figure S17), highlighting the unique photophysical properties imparted by the N‐modified linking units.

To quantitatively analyze the excited‐state dynamics, we employed a state‐to‐state sequential evolution model incorporating five discrete excited‐state species. Global spectral analysis yielded evolution‐associated difference spectra (EADS) and corresponding rate constants (Figure 5c). For C_5_N_1_‐PDI, the first EADS is assigned to the promptly formed CT exciton state. The second EADS, exhibiting a spectral lineshape similar to the first but with markedly diminished amplitude, is also identified as a CT exciton—the amplitude reduction arising from exciton‐exciton annihilation occurring within hundreds of femtoseconds under the experimental excitation density. The third and fourth EADS both display characteristic PDI^2−^ absorption features and are accordingly assigned to the two‐electron accumulated charge‐separated state. The two‐electron accumulation forms with a time constant of 2.11 ps and exhibits biphasic decay with lifetimes of 73 and 937 ps. The fifth EADS corresponds to FC generation, whose dynamics were further characterized by ns‐TA measurements. Remarkably, when C_5_N_1_‐PDI is dispersed in a DMF:H_2_O (1:2 v/v) mixed solution, the CT‐state formation, two‐electron accumulation, and exciton dissociation processes are all significantly accelerated (Figure 5d–f). This enhancement can be attributed to two key factors: (1) the mixed solvent provides a higher dielectric environment that stabilizes charge‐polarized states and promotes efficient charge separation, and (2) water molecules act as reaction substrates that consume photogenerated FCs.

The dynamics of FC generation and recombination, which are crucial for multi‐electron redox catalysis, were examined by monitoring the spectral evolution of the two‐electron accumulated state (characterized by PDI^2^^−^ ESA) on nanosecond timescales. Global analysis of ns‐TA spectra collected over a 1‐µs window reveals that C_5_N_1_‐PDI in DMF:H_2_O mixed solvent undergoes biphasic decay of the two‐electron accumulated state with lifetimes of 0.86 and 22.4 ns (Figure 5g,h). This decay kinetics is substantially faster than those observed for C_4_N_2_‐PDI (2.15 and 20.3 ns) and C_3_N_3_‐PDI (single lifetime of 20.7 ns) under identical conditions (Figures S18 and S19), demonstrating that the asymmetric chemical structure and enhanced BIEF in C_5_N_1_‐ PDI significantly promote charge separation from the multi‐electron accumulated state to photocarriers.

To further understand these effects, we evaluated the reorganization energies between various electronic states through computational analysis of relaxed structures and corresponding energies of dendrimeric PDI fragments (Figure 5i). Exciton diffusion was treated as an excitation energy transfer (EET) process [57], with energy dissipation quantified by the reorganization energy between S_0_ and S_1_ states (λ_EET_ = λ_S0→S1_ + λ_S1→S0_). Exciton dissociation was assessed using combined reorganization energies of electron transfer (λ_ET_ = λ_S0→anion_) and hole transfer (λ_HT_ = λ_S1→anion_) [58]. As illustrated in Figure S20, C_5_N_1_‐PDI exhibits substantially lower reorganization energies for exciton dissociation, indicating superior efficiency in photocarrier generation compared to the other systems [59]. In contrast, all three polymers display similar λ_EET_ values, reflecting comparable exciton diffusion capabilities. Complementing these kinetic insights, we calculated the exciton binding energies (ΔE_e_) to evaluate the thermodynamic feasibility of exciton dissociation. C_5_N_1_‐PDI shows the smallest ΔE_e_ (1.029 eV) among the series (C_4_N_2_‐PDI: 1.087 eV; C_3_N_3_‐PDI: 1.115 eV; C_6_‐PDI: 1.057 eV), which further supports its enhanced charge separation efficiency (Table S5). The thermodynamic driving forces for the formation of PDI˙^−^ and PDI^2^^−^ are further evaluated for the PDI units in C_5_N_1_‐PDI, C_4_N_2_‐PDI, and C_3_N_3_‐PDI (Figure S21). In C_3_N_3_‐PDI, where the PDI units are equivalent, the free energy changes (ΔG) for the formation of PDI˙^−^ and PDI^2^^−^ are −64.52 and 10.24 kcal/mol, respectively (Table S6). For C_4_N_2_‐PDI, the ΔG values for generating PDI˙^−^ and PDI^2^^−^ at the ortho‐PDI (2‐Pym‐PDI) and para‐PDI (4‐Pym‐PDI) sites are −61.62 and −62.88 kcal/mol for the PDI˙^−^, and 13.16 and 10.31 kcal/mol for the PDI^2^^−^, respectively. In comparison, for C_5_N_1_‐PDI, the ΔG values for forming PDI˙^−^ and PDI^2^^−^ at the ortho‐PDI (2‐Py‐PDI) and para‐PDI (4‐Py‐PDI) sites are −61.98 and −64.53 kcal/mol, and 14.41 and 9.26 kcal/mol, respectively (Table S6). These results indicate that the C_5_N_1_‐PDI system is the most thermodynamically favorable for constructing multi‐electron‐enriched states. Moreover, the kinetic advantage observed in C_5_N_1_‐PDI—reflected in the significantly shorter lifetimes of the two‐electron accumulated state (0.86 and 22.4 ns, as shown by ns‐TA)—arises from its enhanced built‐in electric field, which accelerates charge extraction and reduces recombination. Taken together, the favorable exciton binding energy combined with thermodynamically accessible multi‐electron accumulation provides the energetic basis for the superior performance of C_5_N_1_‐PDI.

To directly link the linker chemistry to catalytic activity, we further calculated the free energy profiles for OER on the N sites of the pyridine, pyrimidine, and 1,3,5‐triazine linkers. The OER pathway was modeled as sequential adsorption of H_2_O and formation of ^*^OH, ^*^O, and ^*^OOH intermediates (Figure S22). The calculated free energies for C_5_N_1_‐PDI are 2.47 eV (^*^OH), 3.77 eV (^*^O), and 4.51 eV (^*^OOH). For C_4_N_2_‐PDI and C_3_N_3_‐PDI, the corresponding values are 2.62, 3.64, 4.61 eV and 2.70, 3.64, 3.19 eV, respectively. Notably, C_5_N_1_‐PDI exhibits a more balanced and stepwise free energy profile, particularly with a favorable deprotonation step on the pyridine N, consistent with its higher photocurrent density and stable multi‐electron accumulation observed experimentally (Figure S21). Collectively, these comprehensive spectroscopic and theoretical investigations into excited‐state dynamics, charge separation, multi‐electron accumulation, and catalytic energetics reveal a coherent mechanism linking molecular dipole design to enhanced built‐in electric field, ultrafast charge separation, and efficient photoelectrochemical water oxidation in C_5_N_1_‐PDI.

## 3 Conclusion

This work demonstrates that precise manipulation of nitrogen‐containing linkers in dendrimeric PDI polymers creates tailored molecular dipoles that generate powerful built‐in electric fields, fundamentally governing photoelectrochemical water splitting performance. The superiority of C_5_N_1_‐PDI is rooted in its exceptional reaction kinetics, with ultrafast spectroscopic evidence revealing unprecedented intramolecular charge separation (<2 ps), efficient formation of multi‐electron accumulated states (e.g., PDI^2−^), and significantly accelerated carrier generation. The enhanced built‐in field promotes directional charge transport while reducing recombination losses, enabling photocurrent densities that surpass conventional organic photoanodes. These findings establish a critical structure‐kinetics‐performance relationship, highlighting that molecular dipole engineering represents a versatile strategy for optimizing charge separation dynamics and catalytic efficiency in organic semiconductors for solar fuel generation.

## 4 Experimental Section/Methods

### 4.1 Chemicals and Materials

A total of 2,4,6‐Tribromopyridine (98%), 2,4,6‐trichloropyrimidine (98%), cyanuric chloride (99%), and 3,4,9,10‐perylenetetracarboxylic diimide (98%) were purchased from Bide Pharmtech Ltd. Carbon cloth (CC, WOS1002, 1.5 mm thickness) and indium tin oxide (ITO) coated glass (sheet resistance: 10 Ω sq^−1^) were obtained from CeTech Co., Ltd. 1,8‐Diazabicyclo[5.4.0]undec‐7‐ene (DBU, 99%), tetramethylammonium hydroxide (TMAH, 10 wt.% in MeOH), N,N‐dimethylacetamide (DMAc, 99.8%, anhydrous), dimethyl sulfoxide (DMSO, 99.9%), and methanol (HPLC grade) were supplied by Energy Chemical Co. All chemicals were used as received without further purification. Milli‐Q water (18.2 MΩ·cm) from a Millipore system was used throughout the experiments.

### 4.2 General Procedure for PDI Polymer Synthesis

The PDI polymers were synthesized following a modified literature procedure [39]. In a typical synthesis, SA‐PDI (390 mg, 1.0 mmol), DBU (456 mg, 3.0 mmol), and the respective linking agent (0.7 mmol) were combined with anhydrous DMAc (35 mL) in a three‐neck flask under argon atmosphere. The reaction mixture was stirred at 60°C for 48 h, then quenched with 10 wt.% TMAH aqueous solution (2.0 mL). After cooling to room temperature, the product was collected by filtration through a polyterafluoroethylene (PTFE) membrane (0.22 µm pore size) and sequentially washed with DMSO (3 × 50 mL) and methanol (3 × 50 mL). The final product was dried at 80°C under vacuum overnight.

### 4.3 Synthesis of C_3_N_3_‐PDI

Cyanuric chloride (129 mg, 0.7 mmol) was used as the linking agent. The product was obtained as a dark brown powder (385 mg, 85% yield).

### 4.4 Synthesis of C_4_N_2_‐PDI

A total of 2,4,6‐Trichloropyrimidine (128 mg, 0.7 mmol) was used as the linking agent. The product was obtained as a dark red powder (395 mg, 87% yield).

### 4.5 Synthesis of C_5_N_1_‐PDI

A total of 2,4,6‐Tribromopyridine (221 mg, 0.7 mmol) was used as the linking agent. The product was obtained as a dark purple powder (409 mg, 90% yield).

### 4.6 Structural and Morphological Analysis

Solid‐state ^1^ ^3^C nuclear magnetic resonance (NMR) spectra were recorded on a Bruker Avance Neo 400WB spectrometer. Fourier‐transform infrared (FT‐IR) spectra were obtained using a Bruker Tensor II spectrometer equipped with an attenuated total reflection (ATR) unit. SEM images were acquired on a Zeiss Gemini 300 microscope operating at 5 kV. Atomic force microscopy (AFM) and Kelvin probe force microscopy (KPFM) measurements were performed using an Oxford Instruments Cypher VRS system. X‐ray diffraction (XRD) patterns were collected on a Bruker D8 ADVANCE diffractometer with Cu Kα radiation (λ = 1.5406 Å), scanning from 4° to 80° (2θ) at a rate of 10° min^−1^.

### 4.7 Physicochemical Properties

Nitrogen physisorption isotherms were measured at 77 K using a Micromeritics ASAP 2020 system. Prior to measurement, samples were degassed at 423 K for 8 h. Specific surface areas were calculated using the Brunauer–Emmett–Teller (BET) method, while pore size distributions were derived from the adsorption branches using the Barrett–Joyner–Halenda (BJH) model. Zeta potentials were determined using a Malvern Zetasizer Nano ZS90 analyzer. Thermogravimetric analysis (TGA) was conducted on a Shimadzu TGA‐50 instrument under nitrogen atmosphere with a heating rate of 10°C min^−1^. Elemental analysis (EA) was performed on an Elementar Vario EL cube analyzer.

### 4.8 Spectroscopic Characterization

UV–vis absorption spectra were recorded on a PerkinElmer Lambda 950 spectrophotometer. The fluorescence quantum yield (*Φ*_F_) of the solution was determined by using Rhodamine 6G (R6G, *λ*_ex_ = 488 nm, *Φ*_F_ = 0.95, in ethanol) as a reference [60]. X‐ray photoelectron spectroscopy (XPS) measurements were carried out in a Scienta Omicron Multiprobe system under ultra‐high vacuum (<2 × 10^−10^ mbar) using monochromatic Al Kα radiation (1486.7 eV). Spectra were calibrated against the C 1s peak at 284.8 eV and fitted with Voigt functions (30% Lorentzian, 70% Gaussian) after Shirley background subtraction. Femtosecond time‐resolved transient absorption spectra were measured using a commercial transient absorption spectrometer (Harpia‐TA, Light Conversion). Briefly, fundamental pulses are derived from an amplified femtosecond Ti:sapphire laser (Astrella, Coherent). The laser delivers 40 fs pulses at 1 kHz, and the output is split for white‐light continuum generation and optical pumping. The white‐light continuum is used as a broadband optical probe from the near‐UV to the near‐infrared. It is generated by focusing the fundamental laser beam into a 2 mm thick CaF_2_ plate, which is oriented and continuously shifted in perpendicular directions. The required pumping pulse is obtained by an optical parametric amplifier (TOPAS‐C, Light Conversion). The pump and probe beams were overlapped on a 1 mm thick sample cell, and the included polarization angle was set to the magic angle (54.7°) to record the isotropic response. Transient absorption is calculated from consecutive pump‐on and pump‐poff measurements and averaged over 1000 shots. UV–vis absorption spectra of the samples are measured before and after every measurement in a spectrophotometer. No significant photodegradation was observed. The femtosecond time‐resolved differential absorbance data were analyzed by using the R‐package TIMP software with the graphical interface Glotaran and CarpetView (Light Conversion) [61]. In the global target analysis, the differential absorbances ΔA(*t*, *λ*) are decomposed as a superposition of several principal spectral components *ε*_i_(*λ*) weighed by their concentrations *c_i_* (*t*) [62].

(1) $$\begin{array}{c} \Delta A\;\left(t,\lambda \right)=\sum_{i=1}^{n}c_{i}\left(t\right)\varepsilon_{i}\left(\lambda \right)\; \end{array}$$

The ns‐TA spectra were measured by a commercial spectrometer (Time‐Tech Spectra). The generation of the pump beam is the same as that in fs‐TA. The probe beam was generated from a supercontinuum laser (LEUKOS‐DISCO, French) with the spectral region from 350 to 1800 nm, the repetition rate is 2 kHz, pulse width is 700 ps–1 ns. There is no photodegradation after ns‐TA experiments by checking the steady‐state absorption spectra.

### 4.9 Computational Methods

Density functional theory (DFT) calculations for molecular orbitals and electrostatic potential (ESP) distributions were performed using Gaussian 16 with the RB3LYP functional and 6–31G(d) basis set. All geometry optimizations were conducted in the gas phase. In all the cases, frequency analysis was made after geometry optimization to ensure the convergence to an energy minimum. The electronic excitation analysis and the electrostatic potential (ESP) analysis were conducted by the Multiwfn [63] and VMD program [64].

### 4.10 Fabrication of Working Electrodes

The CC substrate (1.0 × 1.5 cm^2^) was cleaned with distilled water and ethanol, followed by hydrothermal treatment in a distilled water/HNO_3_ mixture (2:1, v/v) at 140°C for 210 min to enhance hydrophilicity and adhesion. For PDI/CC electrodes, 3.0 mg of PDI polymer was dispersed in 3 mL of acetonitrile via sonication; 100 µL of the ink (≈1 mg mL^−^^1^) was drop‐cast onto the pretreated CC and dried at 60°C, yielding a geometric surface area of 0.3 cm^2^ for testing. For PDI/ITO electrodes, 2.0 mg of PDI polymer was dispersed in 2 mL of acetonitrile; 50 µL of the dispersion was spin‐coated onto the conductive side of pre‐cleaned ITO (1 × 2 cm^2^) and dried at 60°C, yielding a geometric surface area of 0.3 cm^2^ for testing.

### 4.11 PEC Measurements

All photoelectrochemical measurements were conducted on a CHI660E electrochemical workstation using a standard three‐electrode configuration: polymer‐modified CC as working electrode, platinum wire as counter electrode, and saturated Ag/AgCl as reference electrode. The electrolyte was a 0.5 m Na_2_SO_4_ aqueous solution (pH 6.8). Simulated solar illumination was provided by a 200 W xenon lamp (Newport) equipped with an AM 1.5G filter (100 mW cm^−2^). Electrochemical impedance spectroscopy (EIS) was measured with a 10 mV AC perturbation over the frequency range of 100 kHz to 1 Hz at 1.23 V vs. RHE. Mott‐Schottky analysis was performed at 1000 Hz. Incident photon‐to‐current efficiency (IPCE) spectra were acquired using a ZAHNER Zennium Pro workstation with a CIMPS‐QE/IPCE module. Evolved gases were quantified by online gas chromatography (GC9200) equipped with a thermal conductivity detector (TCD) and argon carrier gas.

## Funding

The authors gratefully acknowledge financial support from the National Natural Science Foundation of China (No. 52273187 and 22303008), Guangdong Basic and Applied Basic Research Foundation (2023A1515011306 and 2023A1515240077).

## Conflicts of Interest

None of the authors has a conflicts of interest to disclose.

## Supporting information




**Supporting File**: advs75610‐sup‐0001‐SuppMat.docx.

## Data Availability

The data that supports the findings of this study are available in the supplementary material of this article.
