# Supplementary material for: Dipole‐Manipulated Built‐In Electric Field Enables Ultrafast Charge Separation in Perylene Diimide Polymers for Photoelectrochemical Water Splitting
Source: Adv Sci (Weinh). 2026 May 7;13(43):e75610. doi: 10.1002/advs.75610 (PMC13336128; doi:10.1002/advs.75610)
Supplement: Supplementary file 1 — Supporting File: advs75610‐sup‐0001‐SuppMat.docx. [file ADVS-13-e75610-s001.docx]

Supporting Information

Dipole-Manipulated Built-in Electric Field Enables Ultrafast Charge Separation in Perylene Diimide Polymers for Photoelectrochemical Water Splitting

Ying-Xin Qiao, Zicong Situ, Yi-Jing Chen, Shuo-Xiang Liu, Jing-Lan Zhang, Xingqing Li, Luo-Han Xie, Si-Hang Xie, Qing-Xiao Tong,* Andong Xia, Zhuoran Kuang* and Jing-Xin Jian*

Y.X. Qiao, Y.J. Chen, S.X. Liu, J.L. Zhang, L.H. Xie, S.H. Xie, Q.X. Tong, J.X. Jian

Department of Chemistry, Key Laboratory for Preparation and Application of Ordered Structural Materials of Guangdong Province, Shantou University, 515063, Guangdong, P. R. China

E-mail: qxtong@stu.edu.cn; jxjian@stu.edu.cn

Z. Situ, X. Li, A. Xia, Z. Kuang

State Key Laboratory of Information Photonic and Optical Communications and School of Science, Beijing University of Posts and Telecommunications, Beijing 100876, P. R. China

E-mail: kuang@bupt.edu.cn

Y.X. Qiao, Z. Situ and Y.J. Chen contributed equally to this work.


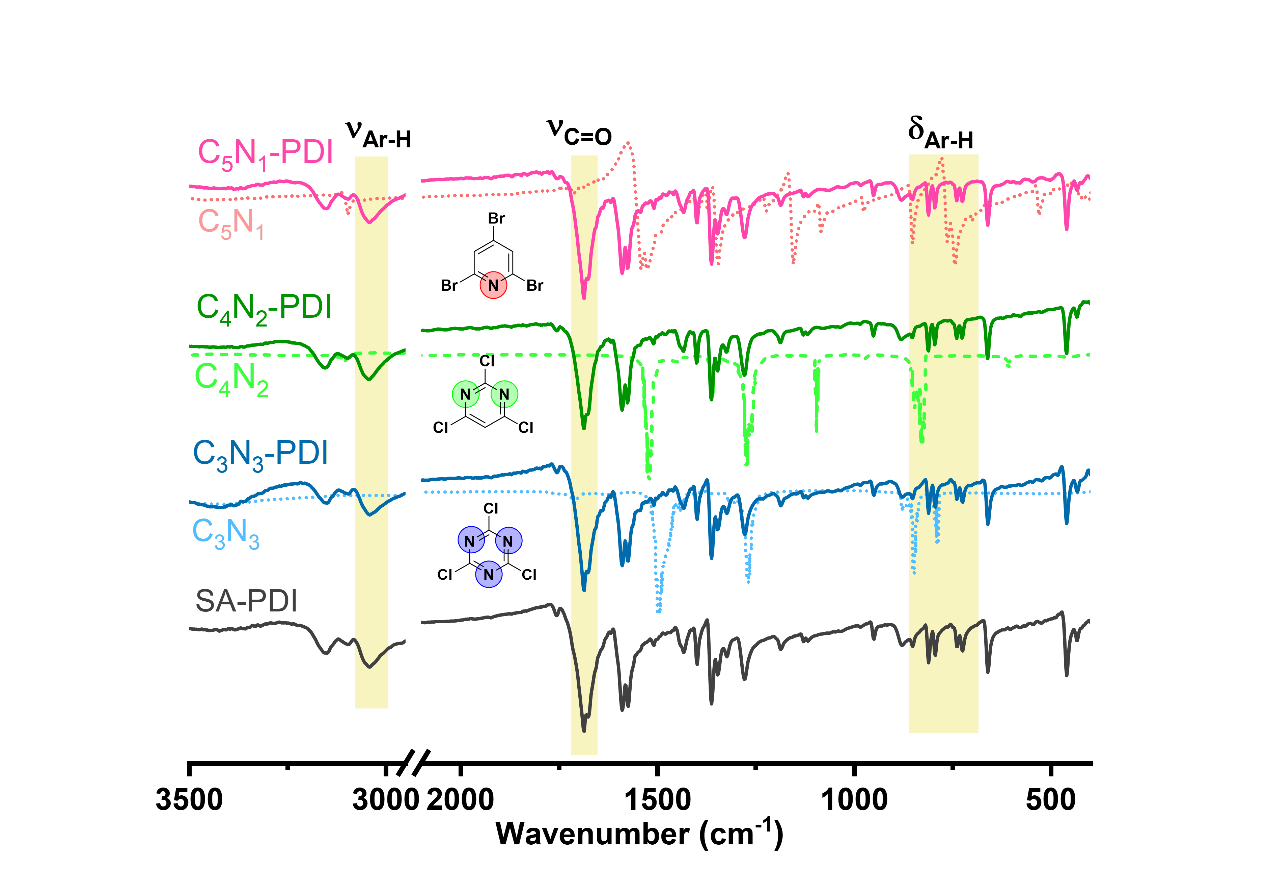


**Figure S1.** FTIR spectra of C_5_N_1_-PDI, C_4_N_2_-PDI, C_3_N_3_-PDI and other reference compounds.


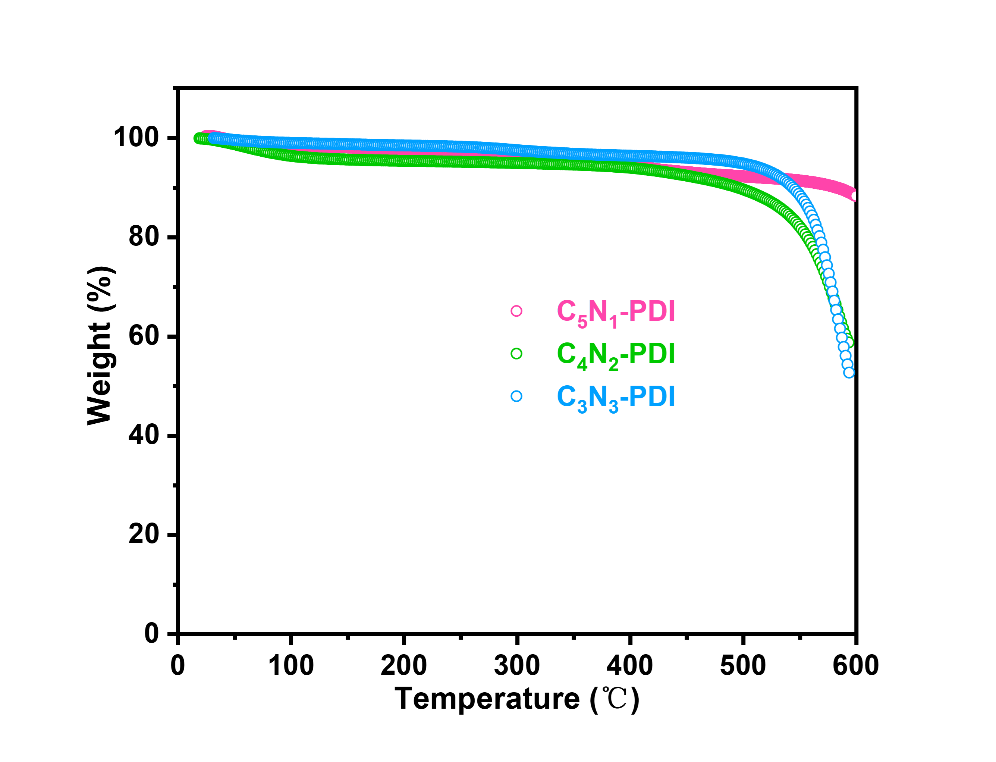


**Figure S2.** TGA of C_5_N_1_-PDI, C_4_N_2_-PDI and C_3_N_3_-PDI.


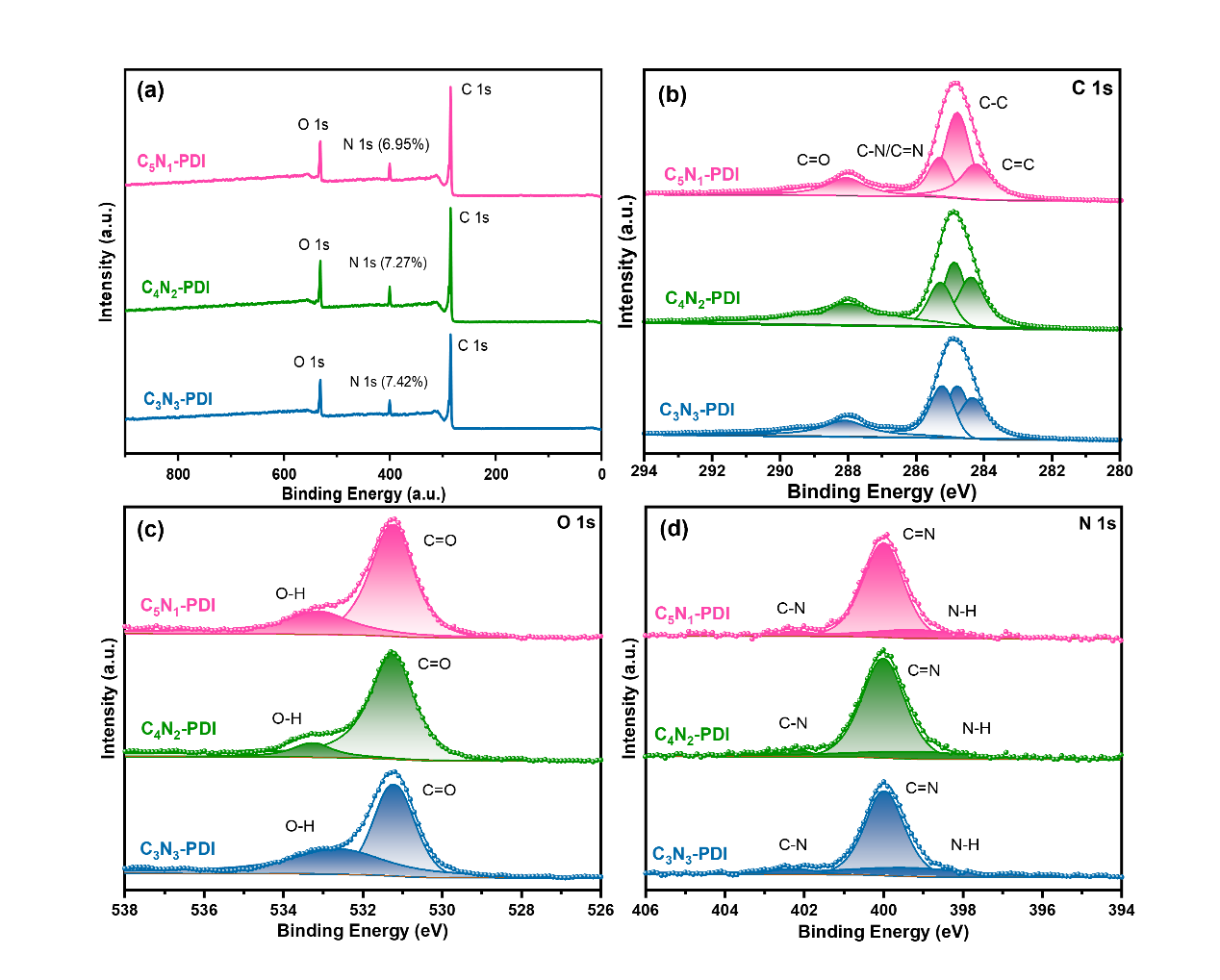


**Figure S3.** XPS survey spectra (a) of C_5_N_1_-PDI, C_4_N_2_-PDI and C_3_N_3_-PDI, and high-resolution XPS spectra of C 1s (b), N 1s (c) and O 1s (d).

**Table S1.** Element contents of C_5_N_1_-PDI, C_4_N_2_-PDI and C_3_N_3_-PDI measured by EA.

|  | C% | H% | N% |
| --- | --- | --- | --- |
| C_5_N_1_-PDI | 75.40 | 4.08 | 7.24 |
| C_4_N_2_-PDI | 73.88 | 4.36 | 9.26 |
| C_3_N_3_-PDI | 72.09 | 4.23 | 11.08 |


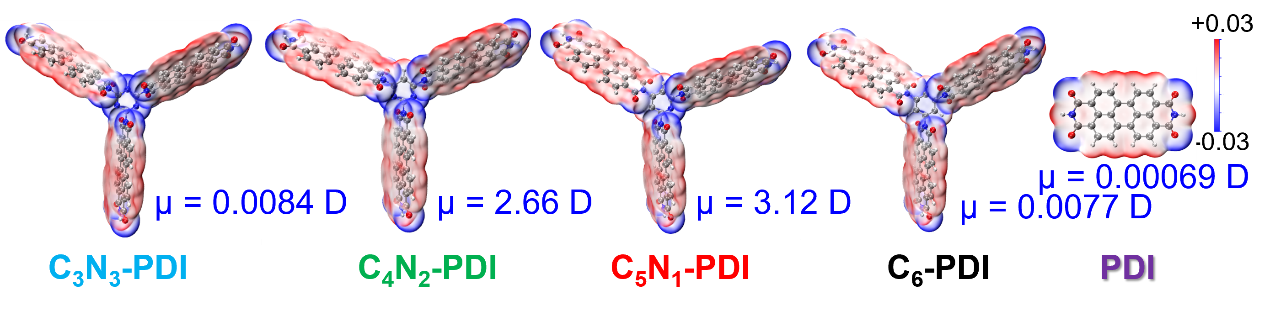


**Figure S4**. DFT-optimized structural models, electrostatic potential (ESP) distribution (blue: electron-rich; red: electron-deficient), and dipole moments (μ) of the different linkers in C_5_N_1_-PDI, C_4_N_2_-PDI, C_3_N_3_-PDI, C_6_-PDI and PDI.


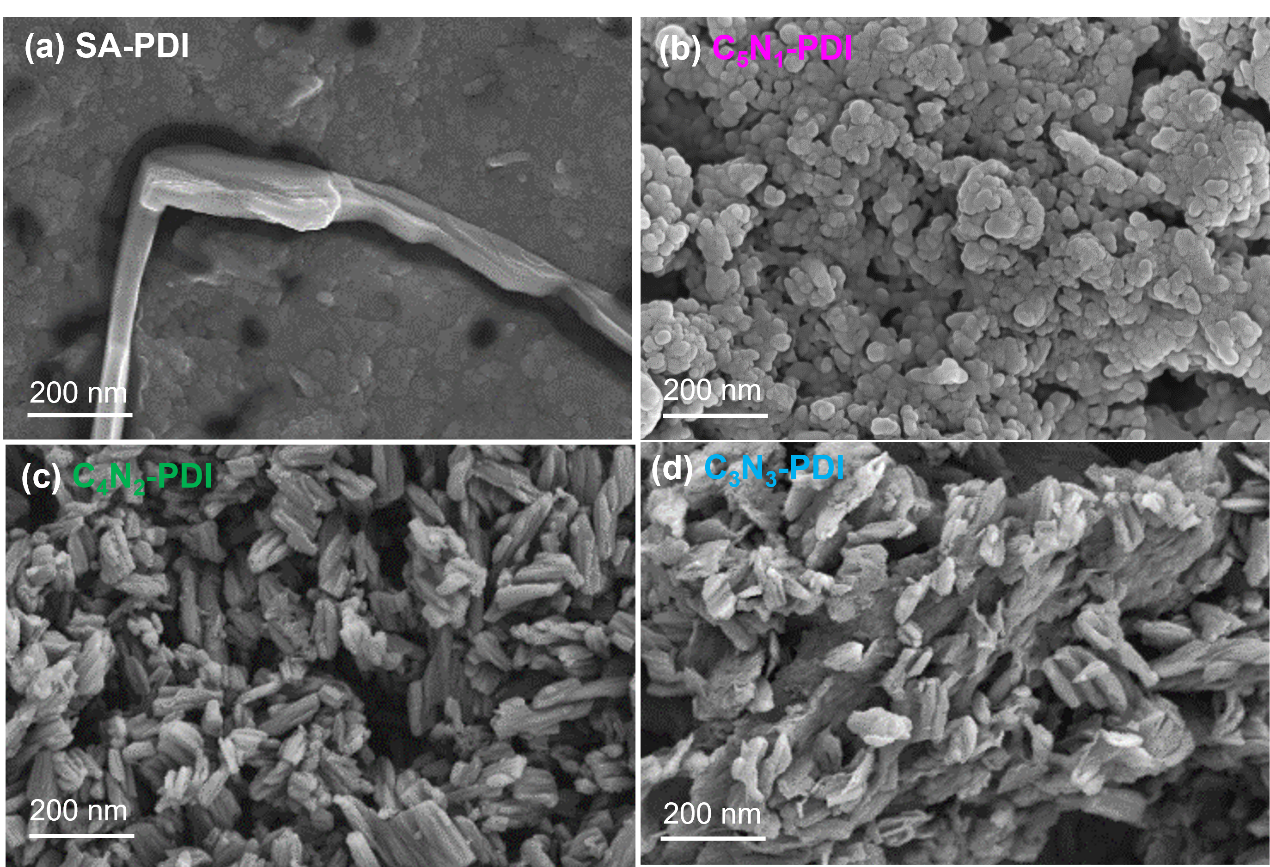


**Figure S5.** FE-SEM images of SA-PDI (a), C_5_N_1_-PDI (b), C_4_N_2_-PDI (c) and C_3_N_3_-PDI (d).


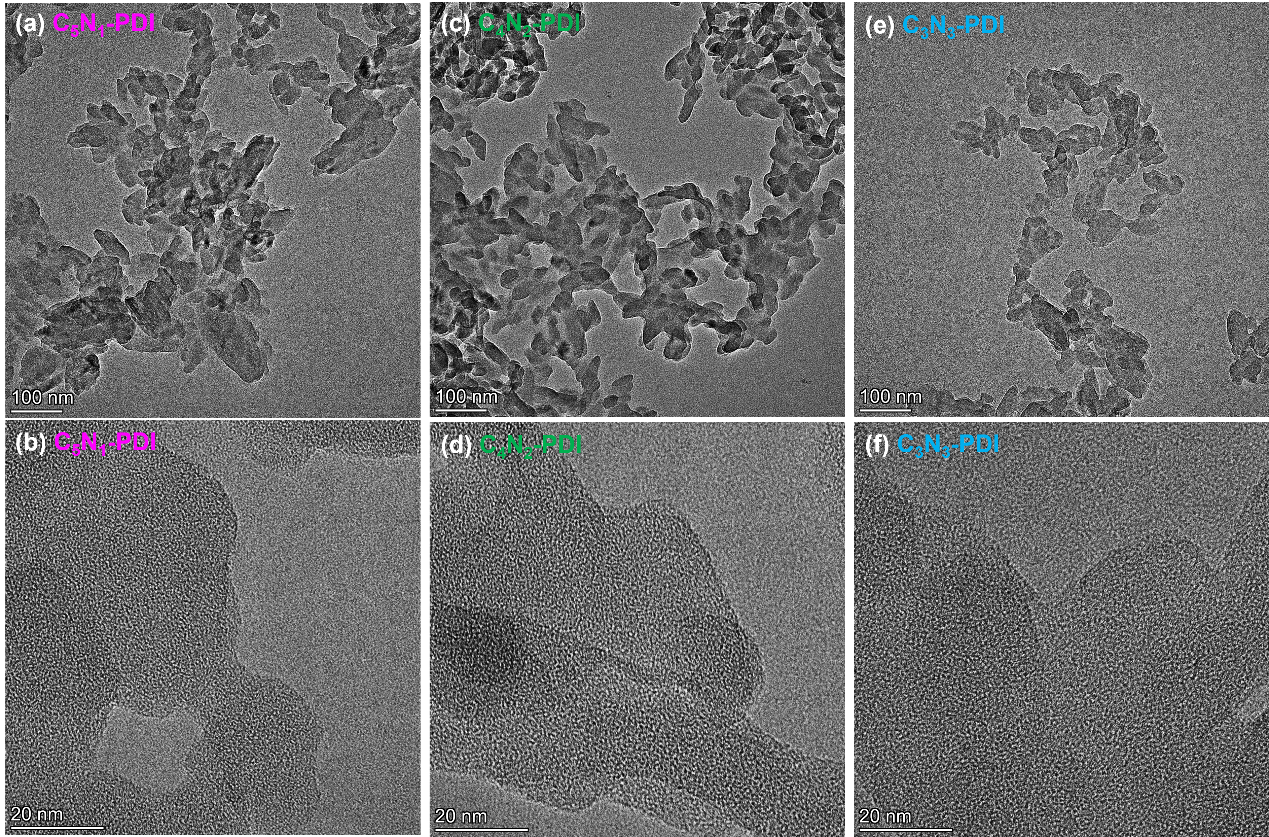


**Figure S6**. High-resolution TEM images of C_5_N_1_-PDI (a,b), C_4_N_2_-PDI (c,d) and C_3_N_3_-PDI (d,e).


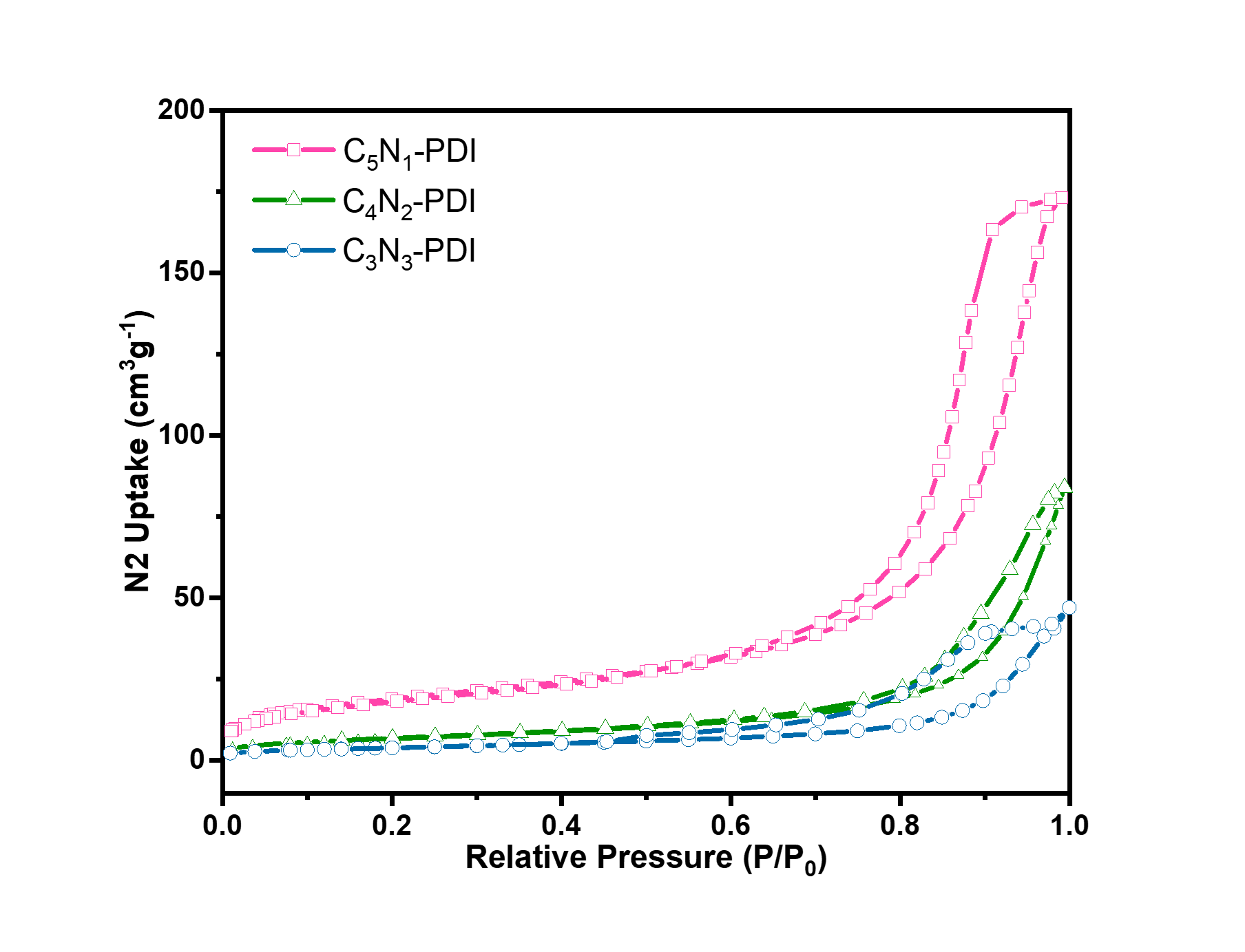


**Figure S7.** N_2_ adsorption-desorption isotherms and pore size distribution curves of C_5_N_1_-PDI, C_4_N_2_-PDI and C_3_N_3_-PDI. The N_2_ adsorption-desorption were established at 77K after the samples were degassed more than 8 h at 423 K. The specific surface area and pore size distribution were calculated by Brunauer-Emmett-Teller (BET) and Barrett-Joyer-Halenda (BJH) equation.

**Table S2.** BET specific surface area and total pore volume.

| Sample | BET surface area (m^2^ g^-1^) | Total pore volume (cm^3^ g^-1^) |
| --- | --- | --- |
| C_5_N_1_-PDI | 77.37 | 0.3527 |
| C_4_N_2_-PDI | 24.50 | 0.1292 |
| C_3_N_3_-PDI | 14.10 | 0.06929 |


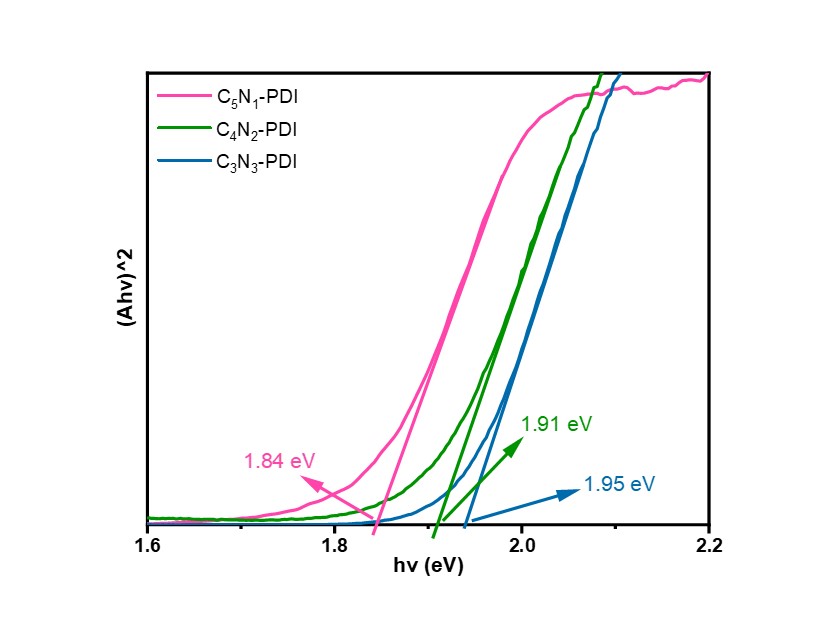


**Figure S8.** Tauc plots of C_5_N_1_-PDI, C_4_N_2_-PDI and C_3_N_3_-PDI.

**Table S3.** Fluorescence quantum yields (*Ф*) of SA-PDI, C_5_N_1_-PDI, C_4_N_2_-PDI, and C_3_N_3_-PDI in DMF.

| Sample | *Ф* |
| --- | --- |
| SA-PDI | 0.99 |
| C_5_N_1_-PDI | 0.01 |
| C_4_N_2_-PDI | <0.01 |
| C_3_N_3_-PDI | 0.01 |

^a^*Ф* with rhodamine 6G as standard (λ_ex_ 488 nm / Φ = 0.95 in ethanol).


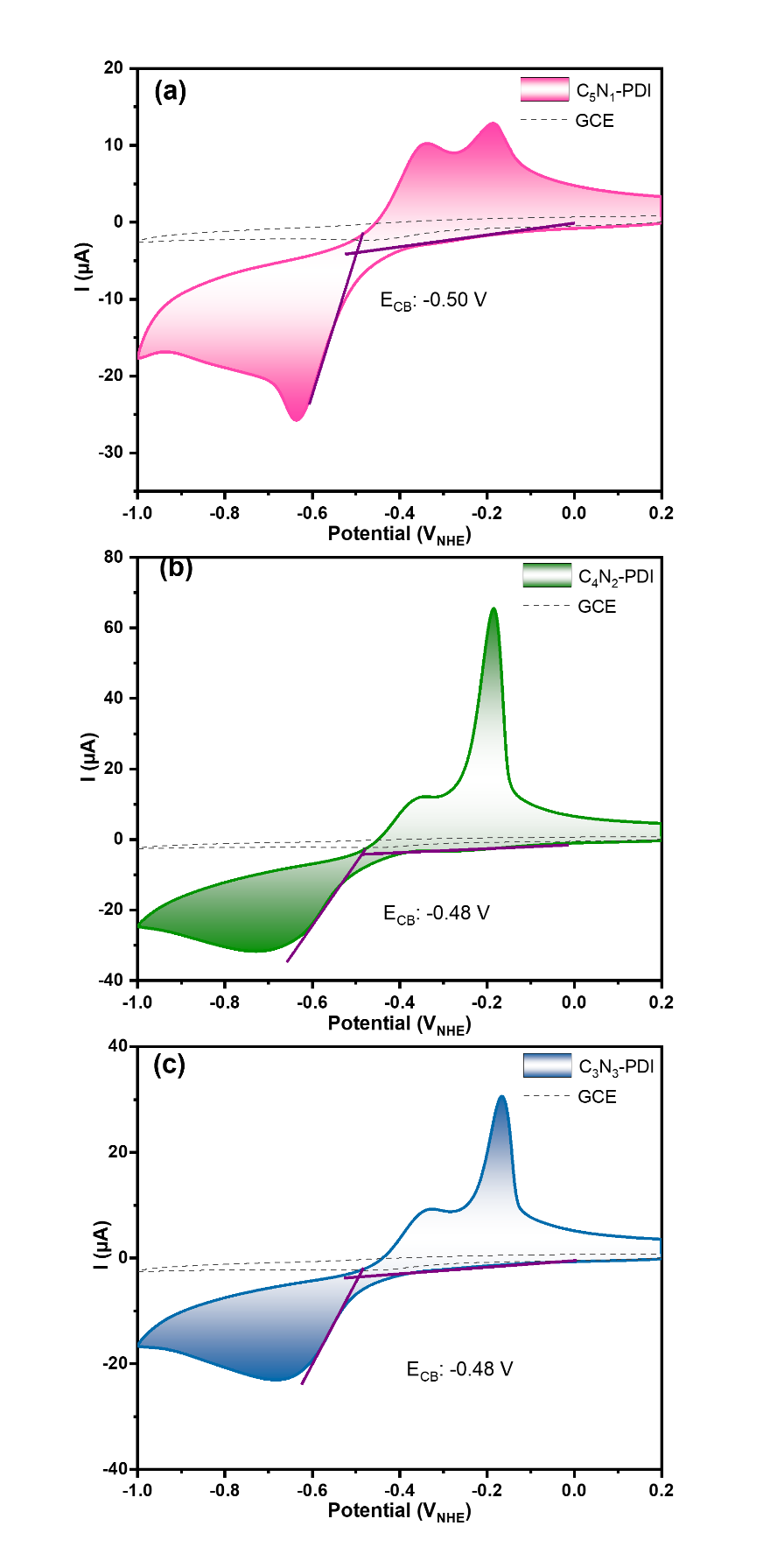


**Figure S9.** CV curves of C_5_N_1_-PDI (a, d), C_4_N_2_-PDI (b, e) and C_3_N_3_-PDI (c, f) in DMF solution containing 0.1 M n-Bu_4_NPF_6_ electrolyte, scan rate: 20 mV s^-1^. The gray dot-line shows the CV curve of glassy carbon electrode (GCE) at the same conditions.


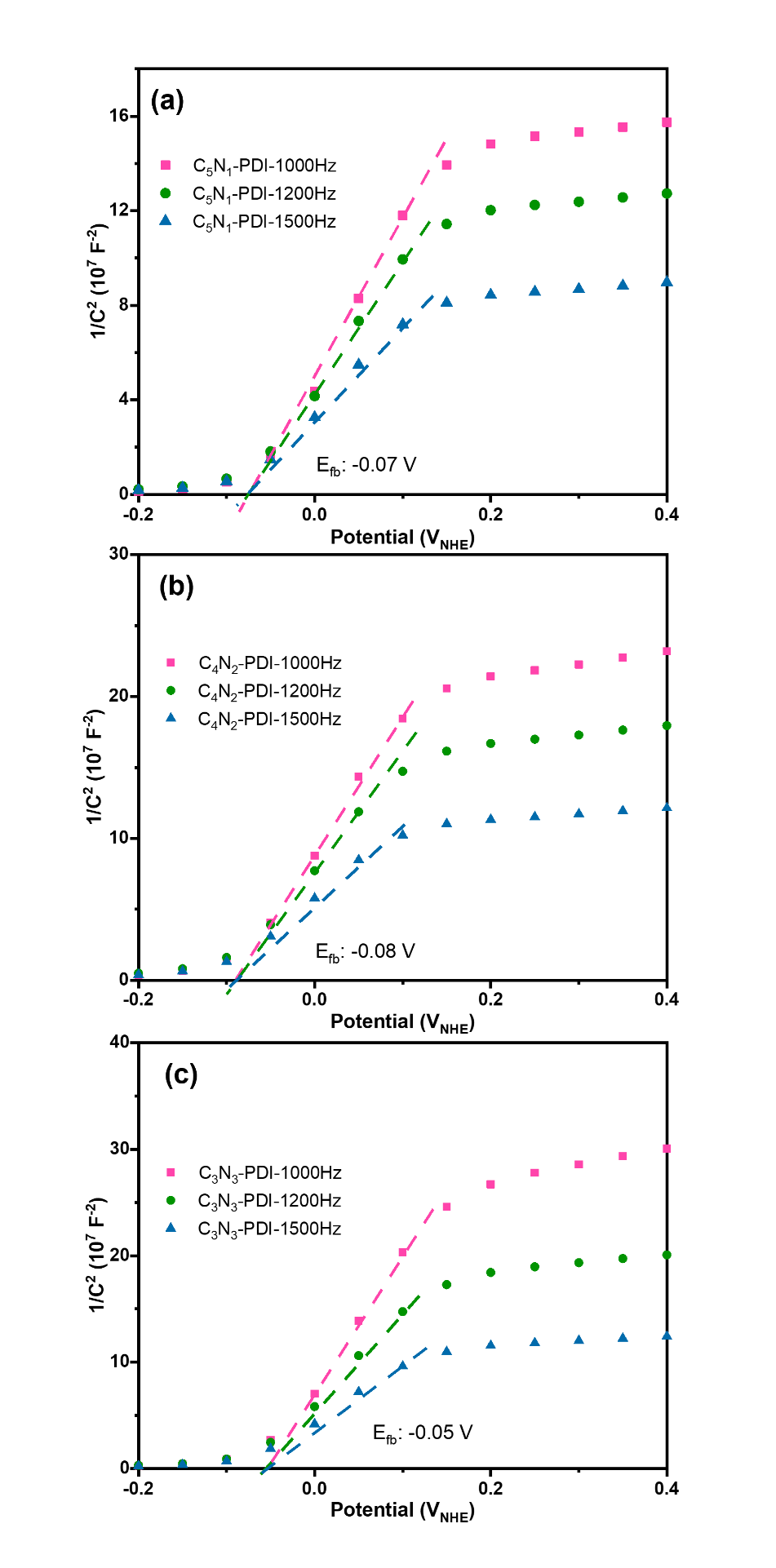


**Figure S10.** Mott-Schottky plots of C_5_N_1_-PDI, C_4_N_2_-PDI and C_3_N_3_-PDI measured in 0.5 M Na_2_SO_4_ aqueous solution (pH 6.8) at a frequency of 1000, 1200 and 1500 Hz.


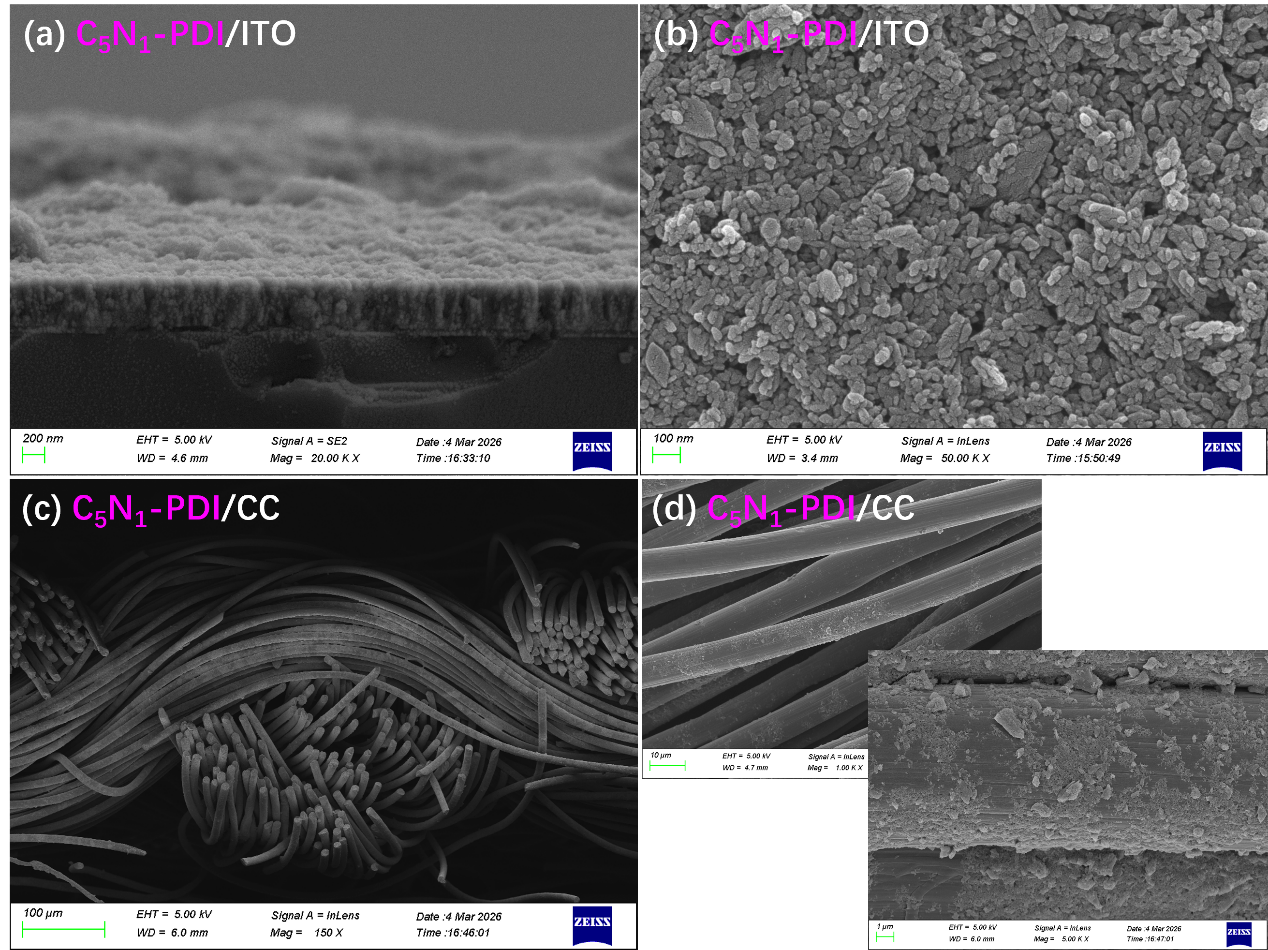


**Figure S11.** Sectional and top-views SEM images of C_5_N_1_-PDI/ITO (a,b) and C_5_N_1_-PDI/CC electrodes.


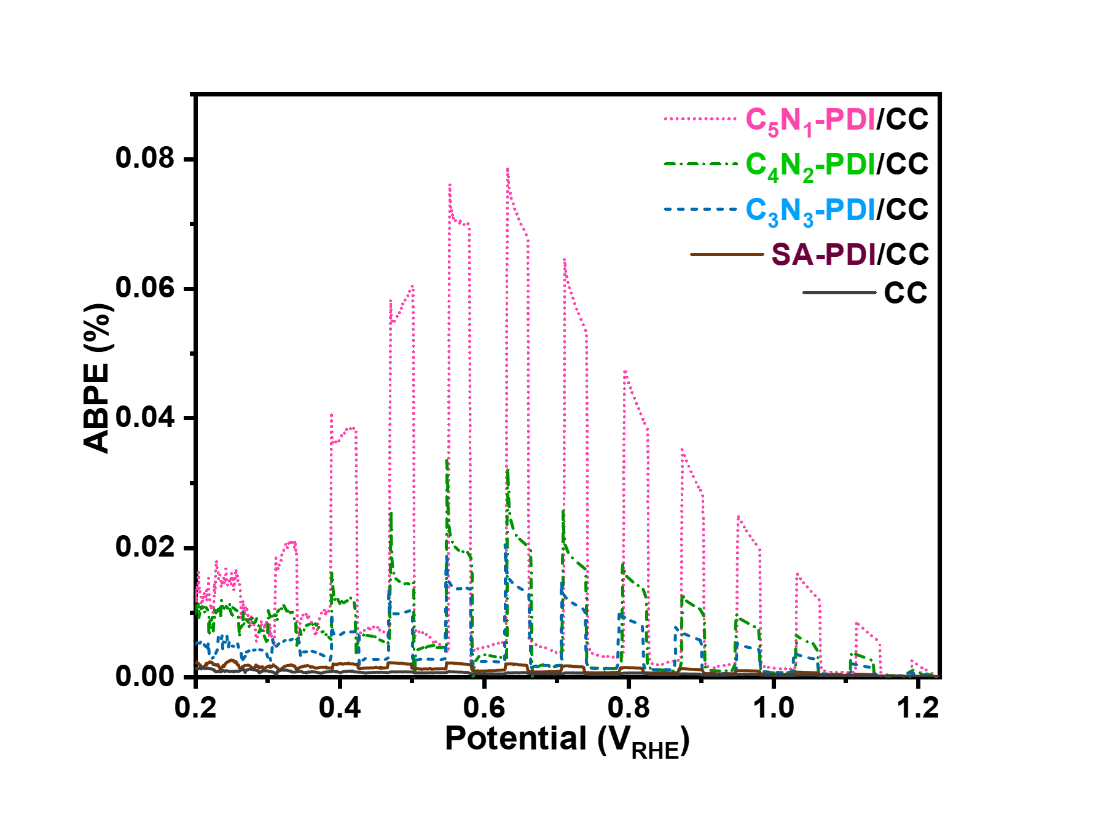


**Figure S12.** ABPEs of C_5_N_1_-PDI/CC, C_4_N_2_-PDI/CC and C_3_N_3_-PDI/CC electrodes.

**Table S4.** Summary of organic-based photoanodes for PEC water oxidation.

| Organic Structure | Electrode | PEC Conditions | PEC Performance | Ref. |
| --- | --- | --- | --- | --- |
|   PDI | IrO_2_/PDI/WO_3_/FTO | AM 1.5G (λ＞435 nm); 0.1 M NaClO_4_ pH 3 | ＜70 μA cm^‑2^,  0.77 V_RHE_ | *J. Am. Chem. Soc.,* **2015**, *137*, 4630.^[1]^ |
|   BBL | Ni-Co/TiO_2_/  BBL/FTO | 100 mW cm^-2^, NaPi pH 7 | ~30 μA cm^-2^,  1.23 V_RHE_ | *J. Am. Chem. Soc*., **2015**, *137*, 15338.^[2]^ |
|   DEMP | IrO_x_+DMEP/  TiO_2_/FTO | λ＞410 nm, 0.1 M NaPi pH 6.8 | ~80 μA cm^-2^,  0.70 V_RHE_ | *PNAS*, **2015**, *112*, 1681.^[3]^ |
| PPor-Oph-COOH | IrWOC1+  PPor-Oph-COOH/  SnO_2_/FTO | 200mW cm^-2^ (λ＞400nm), 0.1 M Na_2_SO_4_ pH 7 | ≤ 20 μA cm^-2^,  0.91 V_RHE_ | *J. Mater. Chem. A*, **2015**, *3*, 3868.^[4]^ |
|   BP | RuWOC+SP/TiO_2_/FTO | LED at 100 mW cm^-2^ (λ＞400 nm), 0.1 M NaF pH 7 | ~800 μA cm^-2^,  0.64 V_RHE_ | *Chem. Commun*., **2016**, *52*, 13702.^[5]^ |
|   PMPDI | CoO_x_/PMPDI/SnO_2_/FTO | ~AM 1.5G (λ＞400nm), 0.1 M NaPi pH 7 | ~20 μA cm^-2^,  1.41 V_RHE_ | *ACS Appl. Mater. Interface*, **2017**, *9*, 27625.^[6]^ |
|   PMI | IrWOC3/Al_2_O_3_/PMI/TiO_2_/FTO | 200mW cm^-2^ (λ＞410nm), 0.1 M Na_2_SO_4_ pH 2.5 | ~170 μA cm^-2^, 0.66 V_RHE_ | *J. Phys. Chem. C*, **2017**, *121*, 3752.^[7]^ |
|   KuQ(O)_3_OH | Ru_4_POM (1% Nafion)/KuQ(O)_3_OH/SnO_2_/FTO | AM 1.5G  (λ＞400 nm),  0.1 M Na_2_SiF_6_-NaHCO_3_ buffer pH 5.8 | ~23 μA cm^-2^,  1.14 V_RHE_ | *Chem. Commun*., **2020**, *56*, 2248.^[8]^ |
|   Urea-PDI | Urea-PDI/ITO | λ＞420nm, Na_2_SO_4_ solution | ~2.1 μA cm^-2^ | *Adv. Mater*., **2020**, *32*, 1907746.^[9]^ |
|   H_2_PDI | Co-PDI (0.5% Nafion)/ITO | 300 W Xe lamp (λ＞420 nm), 0.5 M Na_2_SO_4_ solution | ~0.4 μA cm^-2^ | *Appl. Catal. B Environ*, **2020**, *260*, 118135.^[10]^ |
|   PDI | PDI/Co_3_O_4_/Pt/ITO | 300 W Xe lamp (λ＞420 nm), 0.1 M Na_2_SO_4_ pH 6.8 | ~4 μA cm^-2^ | *ACS Energy Lett*. **2023**, *8*, 2652.^[11]^ |
|   PDI-PDA | PDI-PDI (0.5%Nafion)/Carbon Paper | 400 mW cm^2^ White light,  0.1 M Na_2_SO_4_ solution | ~0.3 μA cm^-2^ | *Angew. Chem. Int. Ed*. **2024**, *63*, e202412977.^[12]^ |
|   oPDI | oPDI/ITO | 100 mW cm^-2^ AM 1.5G, 0.5 M Na_2_SO_4_ pH 6.8 | 115.1 μA cm^-2^  1.4 V_RHE_ | *Angew. Chem. Int. Ed*. **2024**, *63*, e202318224.^[13]^ |
|   C_5_N_1_-PDI | C_5_N_1_-PDI/CC | 100 mW cm^-2^ AM 1.5G, 0.5 M Na_2_SO_4_ pH 6.8 | 68.7 μA cm^-2^  1.23 V_RHE_ | This work |


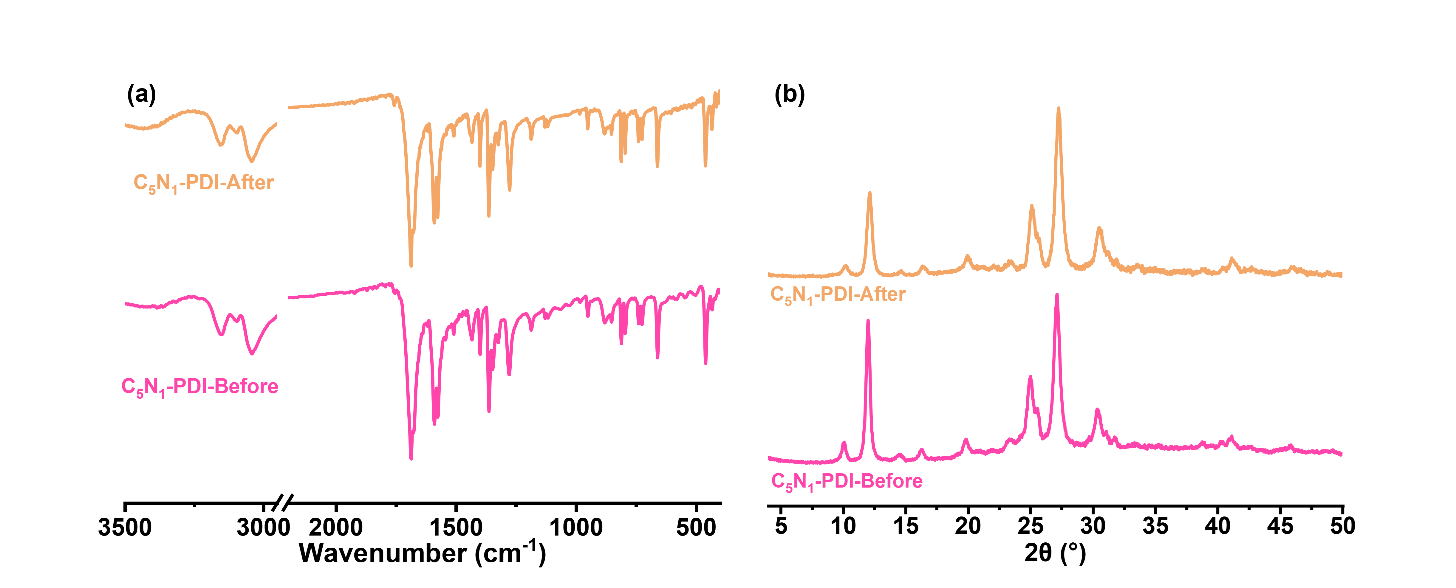


**Figure S13.** FTIR (a) and PXRD (b) of C_5_N_1_-PDI after PEC measurements.


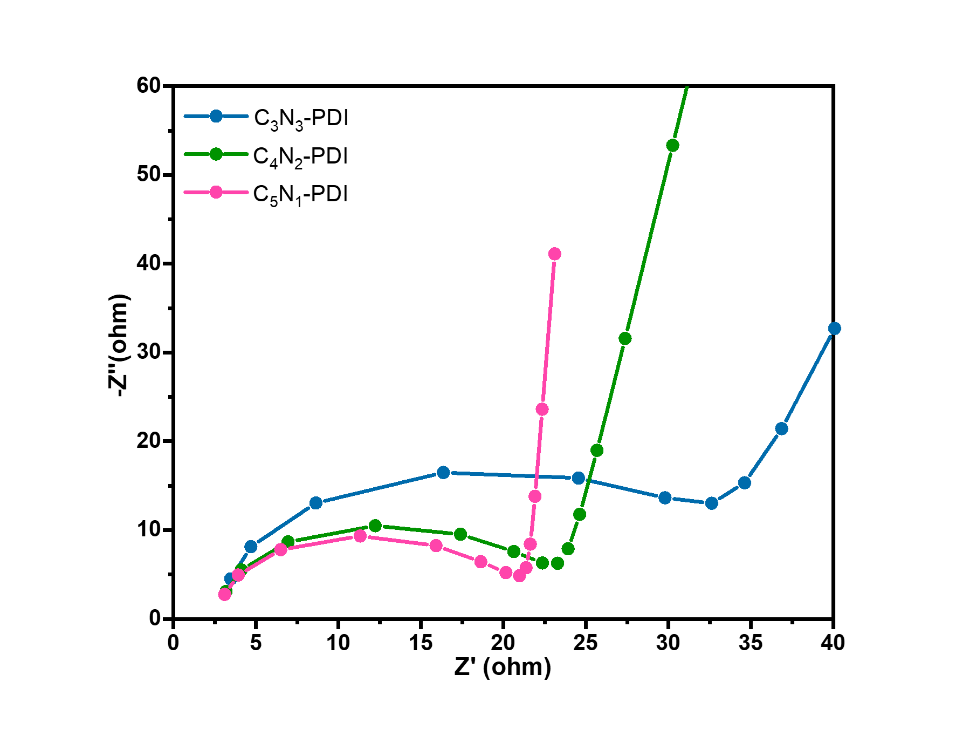


**Figure S14.** EIS of C_5_N_1_-PDI, C_4_N_2_-PDI and C_3_N_3_-PDI electrode at applied potential of 1.23 V_RHE_, under AM1.5G illumination, in 0.5 M Na_2_SO_4_ aqueous solution (pH 6.8).


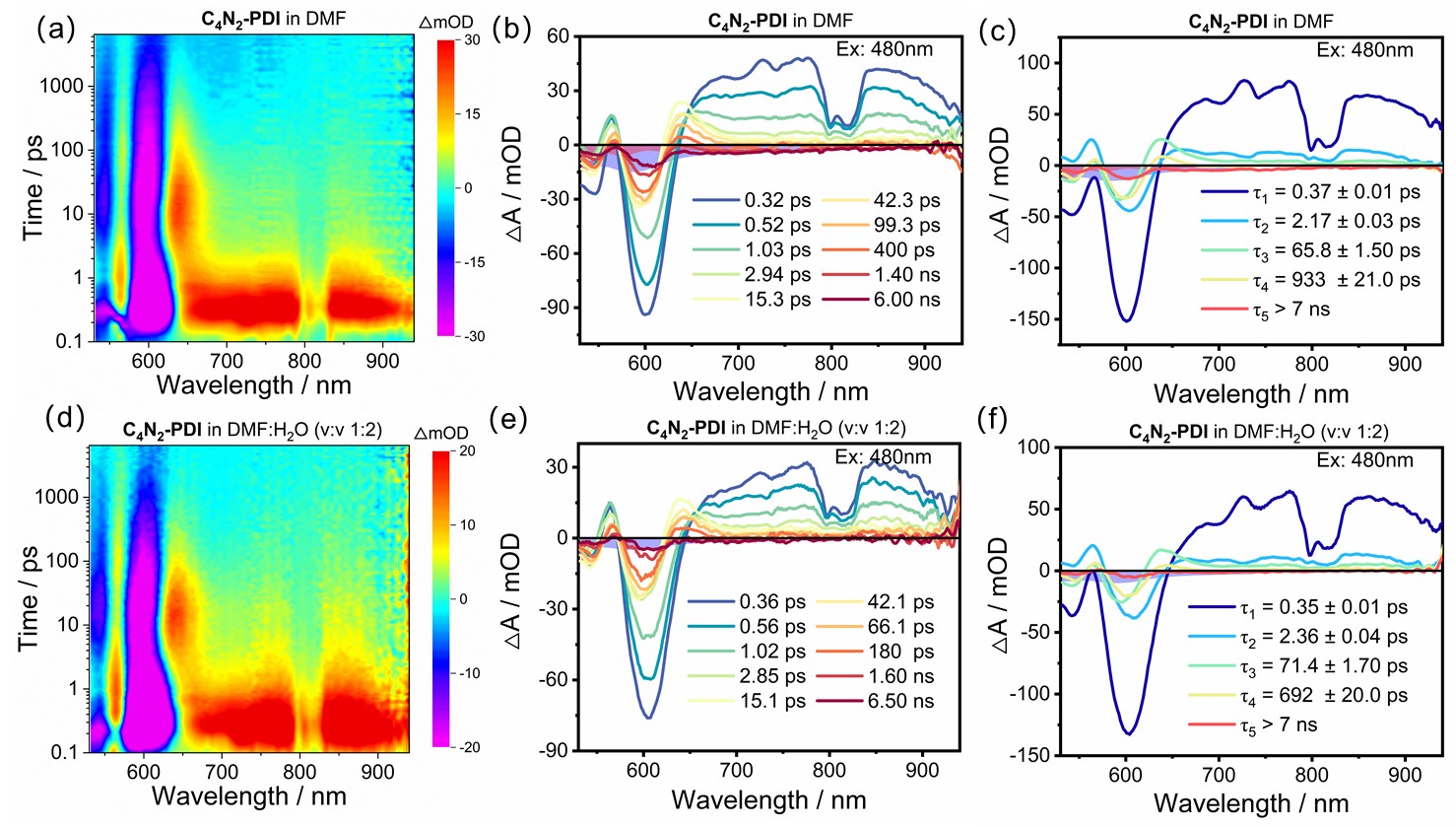


**Figure S15.** 2D pseudocolor plots of fs-TA spectraof C_4_N_2_-PDI in (a) DMF and (d) DMF:H_2_O (v:v 1:2) solution upon excitation at 480 nm. The corresponding time evolution of the fs-TA spectra of C_4_N_2_-PDI in (b) DMF and (e) DMF:H_2_O (v:v 1:2) solution. Evolution-associated difference spectra (EADS) obtained from the global analysis based on a sequential model are shown in the (c) DMF and (f) DMF:H_2_O (v:v 1:2) solution.; The blue and red shaded areas represent the corresponding scaled stationary absorption and emission spectra, respectively, for the identification of GSB and SE signals in the TA spectra.


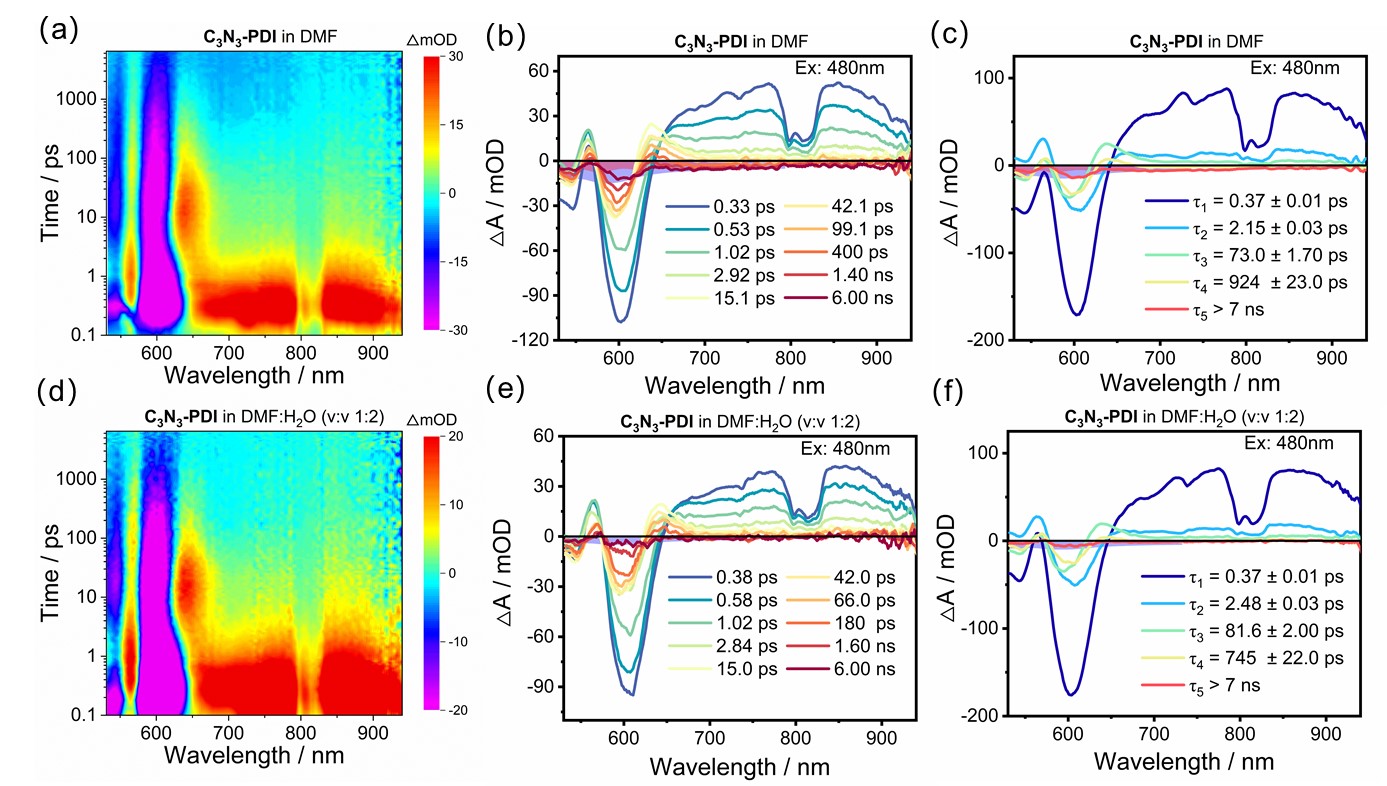


**Figure S16.** 2D pseudocolor plots of fs-TA spectraof C_3_N_3_-PDI in (a) DMF and (d) DMF:H_2_O (v:v 1:2) solution upon excitation at 480 nm. The corresponding time evolution of the fs-TA spectra of C_3_N_3_-PDI in (b) DMF and (e) DMF:H_2_O (v:v 1:2) solution. Evolution-associated difference spectra (EADS) obtained from the global analysis based on a sequential model are shown in the (c) DMF and (f) DMF:H_2_O (v:v 1:2) solution.; The blue and red shaded areas represent the corresponding scaled stationary absorption and emission spectra, respectively, for the identification of GSB and SE signals in the TA spectra.


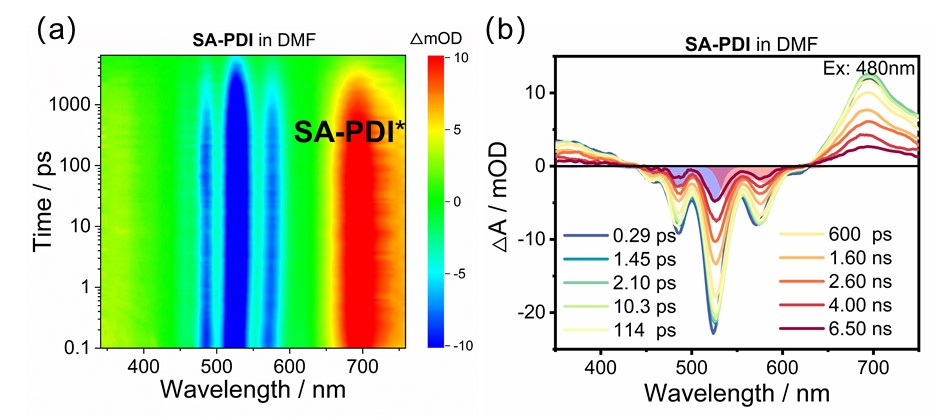


**Figure S17.** (a) 2D pseudocolor plots of fs-TA spectra of SA-PDI in DMF upon excitation at 480 nm. (b) The corresponding time evolution of the fs-TA spectra of SA-PDI in DMF. The blue and red shaded areas represent the corresponding scaled stationary absorption and emission spectra, respectively, for the identification of GSB and SE signals in the TA spectra.


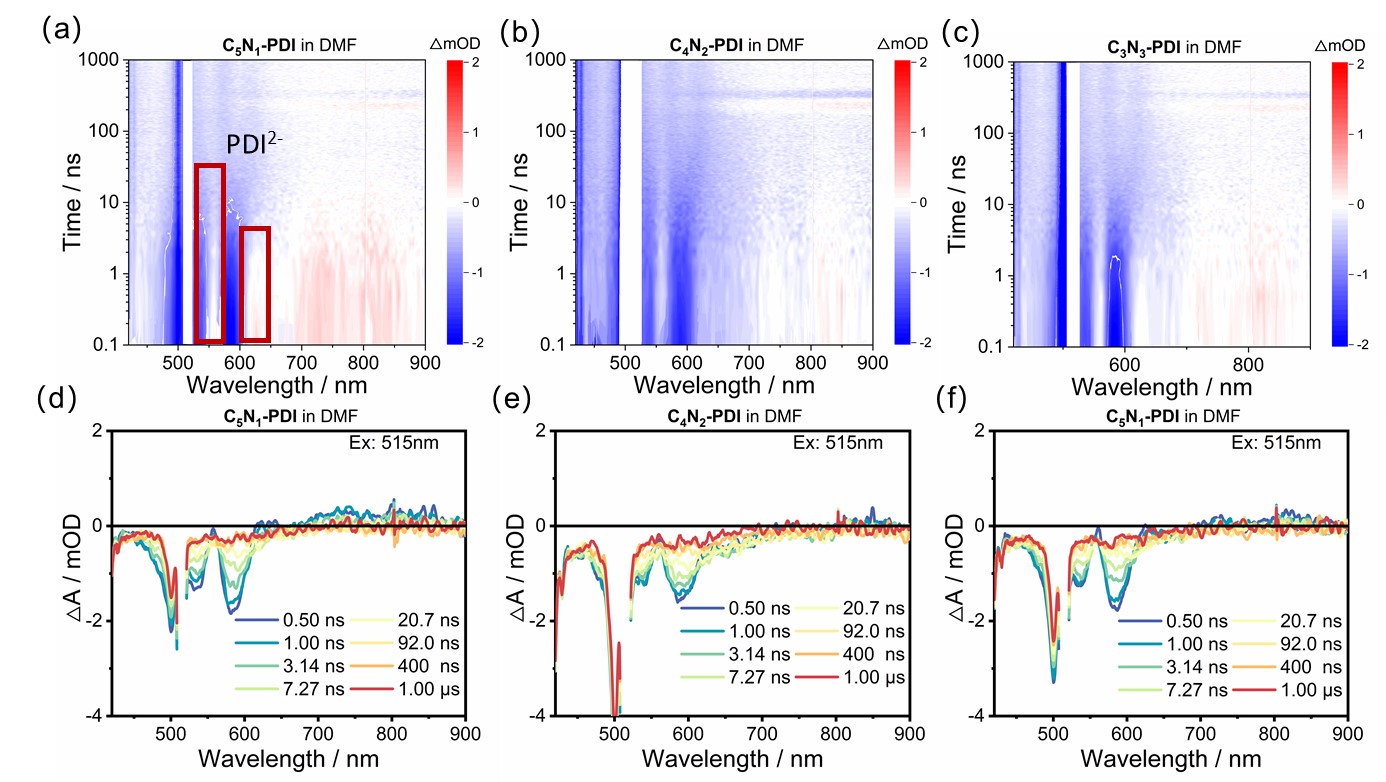


**Figure S18.** 2D pseudocolor plots of fs-TA spectra of (a) C_5_N_1_-PDI, (b) C_4_N_2_-PDI and (c) C_3_N_3_-PDI in DMF solution upon excitation at 515 nm. The corresponding time evolution of the fs-TA spectra of (d) C_5_N_1_-PDI, (e) C_4_N_2_-PDI and (f) C_3_N_3_-PDI in DMF solution.


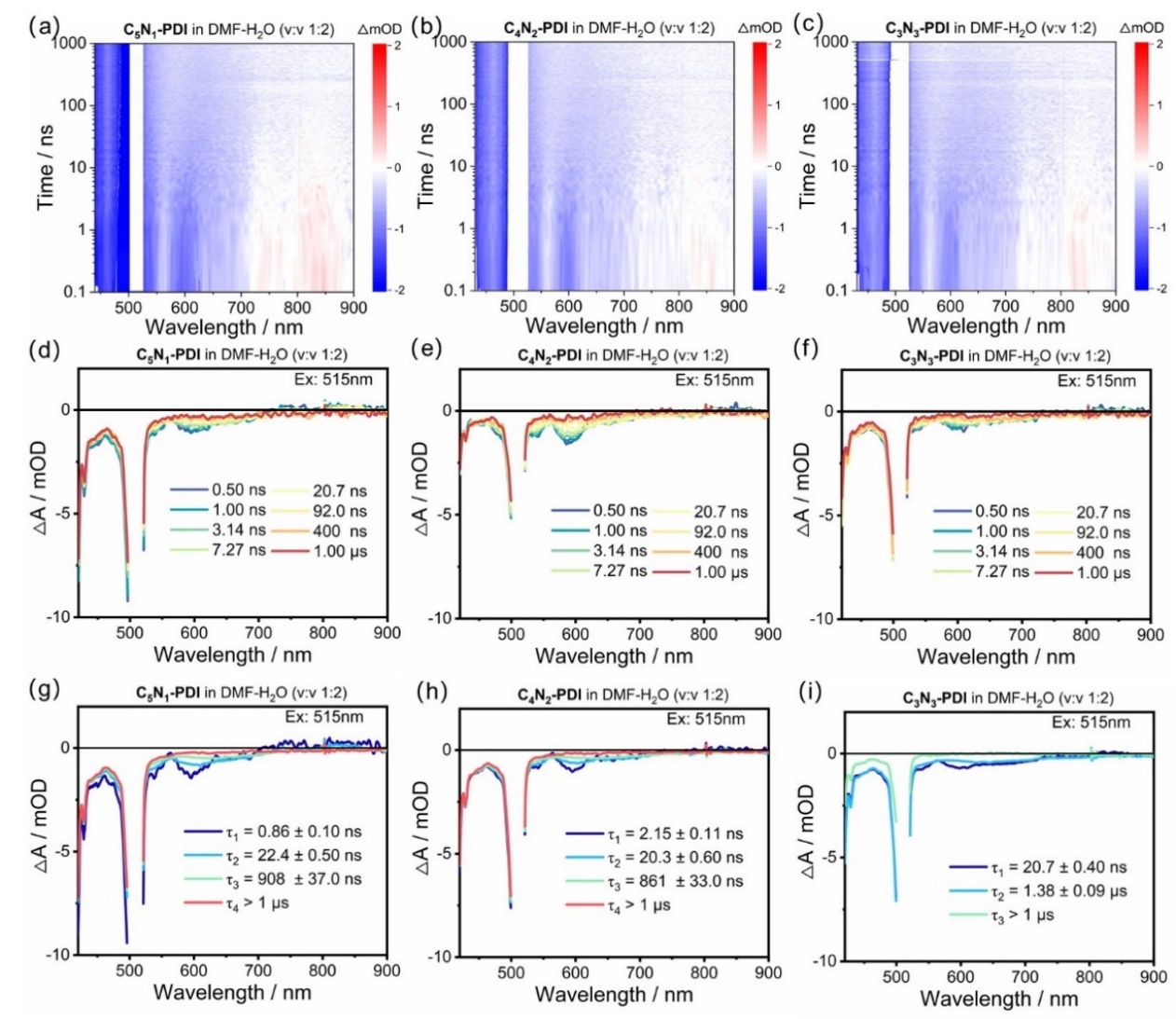


**Figure S19.** 2D pseudocolor plots of fs-TA spectra of (a) C_5_N_1_-PDI, (b) C_4_N_2_-PDI and (c) C_3_N_3_-PDI in DMF:H_2_O (v:v 1:2) solution upon excitation at 515 nm. The corresponding time evolution of the fs-TA spectra of (d) C_5_N_1_-PDI, (e) C_4_N_2_-PDI and (f) C_3_N_3_-PDI in DMF:H_2_O (v:v 1:2) solution. EADS for (g) C_5_N_1_-PDI, (h) C_4_N_2_-PDI and (i) C_3_N_3_-PDI.


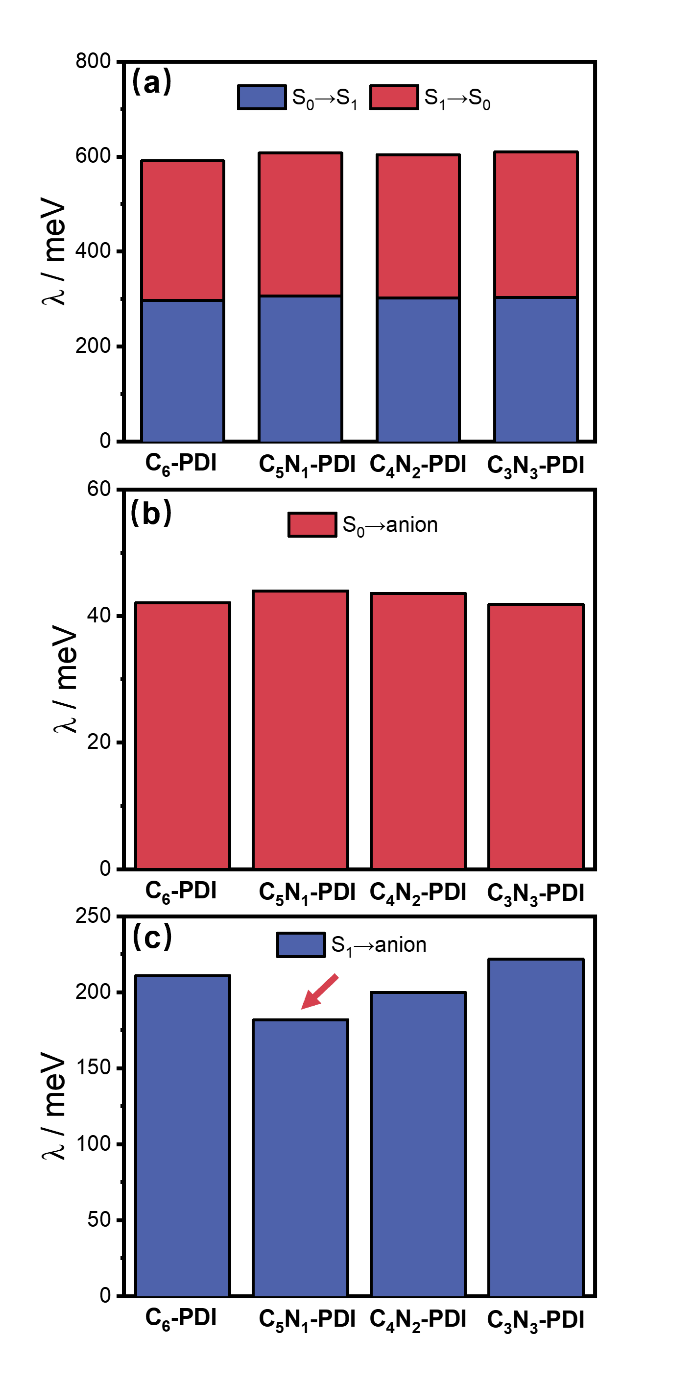


**Figure S20.** Calculated reorganization energies for (a) excitation energy transfer, (b) electron transfer, and (c) hole transfer.

**Table S5.** Calculated exciton binding energies (ΔEₑ) of C_6_-PDI, C_5_N_1_-PDI, C_4_N_2_-PDI and C_3_N_3_-PDI.

| Sample | ΔEₑ (eV) |
| --- | --- |
| C_6_-PDI | 1.057 |
| C_5_N_1_-PDI | 1.029 |
| C_4_N_2_-PDI | 1.087 |
| C_3_N_3_-PDI | 1.115 |


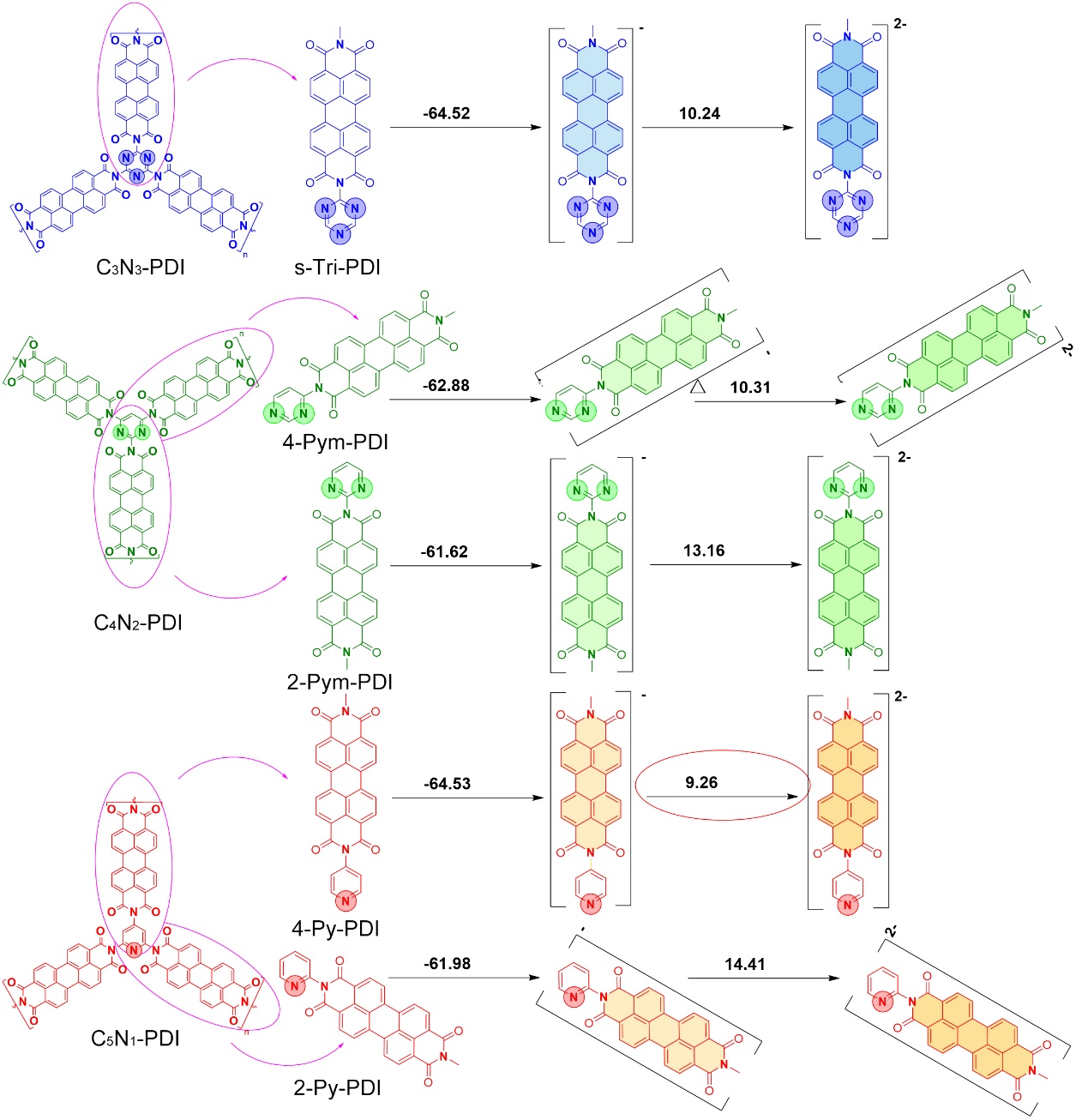


**Figure S21**. The free energy changes of PDI derivatives from PDI to PDI^-^and PDI^2-^at different positions (kcal/mol).

**Table S6**. Free energy of the formation of PDI^-^ and PDI^2-^.

|  | PDI unit | PDI^-^ | PDI^2-^ | | 2PDI^-^ 🡪 PDI + PDI^2-^ | | |
| --- | --- | --- | --- | --- | --- | --- | --- |
| C_5_N_1_-PDI | 2-Py-PDI | -61.98 | 14.41 | 76.39 | | 71.24 (Min.) |  |
|  | 4-Py-PDI | -64.53 | 9.26 | 73.79 | |  |  |
| C_4_N_2_-PDI | 2-Pym-PDI | -61.62 | 13.16 | 74.78 | | 71.93 (Min.) |  |
|  | 4-Pym-PDI | -62.88 | 10.31 | 73.19 | |  |  |
| C_3_N_3_-PDI | s-Tri-PDI | -64.52 | 10.24 | 74.76 | | 74.76 (Min.) |  |

^*^All data in kcal/mol.


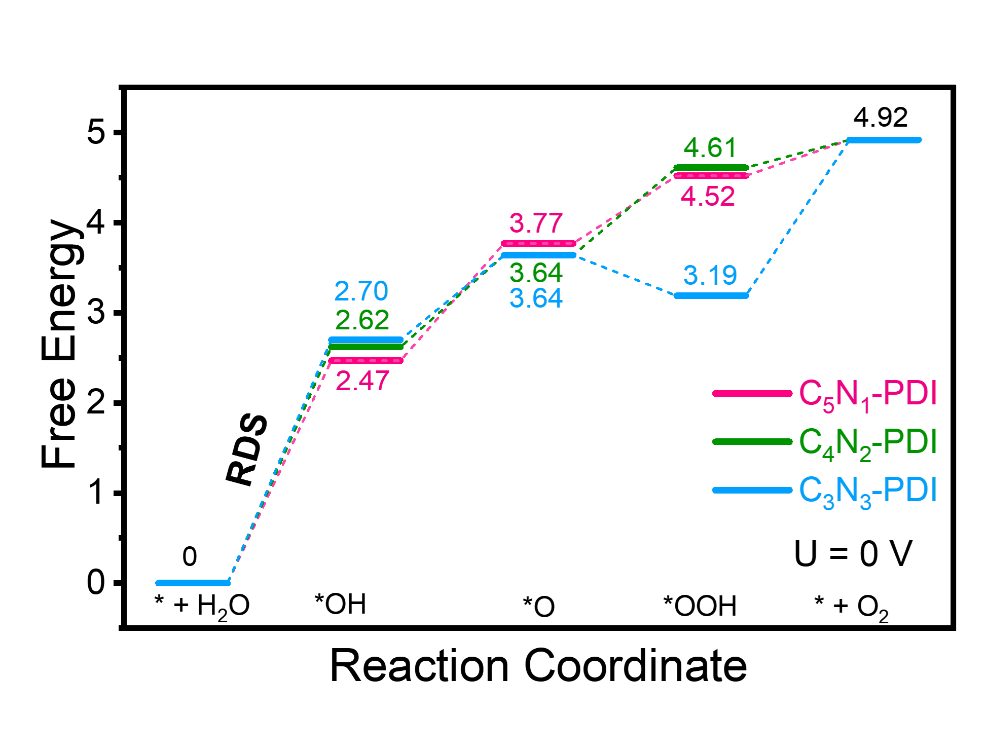


**Figure S22**. Calculated free energy diagrams for OER of C_5_N_1_-PDI, C_4_N_2_-PDI and C_3_N_3_-PDI.

**References**

[1] F. Ronconi, Z. Syrgiannis, A. Bonasera, M. Prato, R. Argazzi, S. Caramori, V. Cristino, C. A. Bignozzi, *J. Am. Chem. Soc.* **2015**, *137*, 4630-4633.

[2] P. Bornoz, M. S. Prevot, X. Yu, N. Guijarro, K. Sivula, *J. Am. Chem. Soc.* **2015**, *137*, 15338.

[3] J. R. Swierk, D. D. Mendez-Hernandez, N. S. McCool, P. Liddell, Y. Terazono, I. Pahk, J. J. Tomlin, N. V. Oster, T. A. Moore, A. L. Moore, D. Gust, T. E. Mallouk, *Proc. Nat. Acad. Sci. USA* **2015**, *112*, 1681.

[4] P. K. Poddutoori, J. M. Thomsen, R. L. Milot, S. W. Sheehan, C. F. A. Negre, V. K. R. Garapati, C. A. Schmuttenmaer, V. S. Batista, G. W. Brudvig, A. van der Est, *J Mater Chem A* **2015**, *3*, 3868.

[5] M. Yamamoto, Y. Nishizawa, P. Chábera, F. Li, T. Pascher, V. Sundström, L. Sun, H. Imahori, *Chem. Commun.* **2016**, *52*, 13702-13705.

[6] J. T. Kirner, R. G. Finke, *ACS Appl. Mater. Interfaces* **2017**, *9*, 27625.

[7] R. J. Kamire, K. L. Materna, W. L. Hoffeditz, B. T. Phelan, J. M. Thomsen, O. K. Farha, J. T. Hupp, G. W. Brudvig, M. R. Wasielewski, *J. Phys. Chem. C* **2017**, *121*, 3752-3764.

[8] G. A. Volpato, M. Marasi, T. Gobbato, F. Valentini, F. Sabuzi, V. Gagliardi, A. Bonetto, A. Marcomini, S. Berardi, V. Conte, M. Bonchio, S. Caramori, P. Galloni, A. Sartorel, *Chem. Commun.* **2020**, *56*, 2248-2251.

[9] Z. J. Zhang, X. J. Chen, H. J. Zhang, W. X. Liu, W. Zhu, Y. F. Zhu, *Adv. Mater.* **2020**, *32*, 1907746.

[10] Z. Zhong, R. Li, W. Lin, X. Xu, X. Tian, X. Li, X. Chen, L. Kang, *App. Catal. B: Environ.* **2020**, *260*, 118135.

[11] W. Li, Z. Wei, Y. Sheng, J. Xu, Y. Ren, J. Jing, J. Yang, J. Li, Y. Zhu, *ACS Energy Lett.* **2023**, *8*, 2652-2660.

[12] Z. Li, J. Jiao, W. Fu, K. Gao, X. Peng, Z. Wang, H. Zhuo, C. Yang, M. Yang, G. Chang, L. Yang, X. Zheng, Y. Yan, F. Chen, M. Zhang, Z. Meng, X. Shang, *Angew. Chem. Int. Ed.* **2024**, *63*, e202412977.

[13] Y. J. Chen, J. Z. Zhang, Z. X. Wu, Y. X. Qiao, L. Zheng, F. Wondu Dagnaw, Q. X. Tong, J. X. Jian, *Angew. Chem. Int. Ed.* **2024**, *63*, e202318224.
